# Dermal and muscle fibroblasts and skeletal myofibers survive chikungunya virus infection and harbor persistent RNA

**DOI:** 10.1371/journal.ppat.1007993

**Published:** 2019-08-29

**Authors:** Alissa R. Young, Marissa C. Locke, Lindsey E. Cook, Bradley E. Hiller, Rong Zhang, Matthew L. Hedberg, Kristen J. Monte, Deborah J. Veis, Michael S. Diamond, Deborah J. Lenschow

**Affiliations:** 1 Department of Molecular Microbiology, Washington University School of Medicine, St. Louis, Missouri, United States of America; 2 Department of Pathology and Immunology, Washington University School of Medicine, St. Louis, Missouri, United States of America; 3 Department of Medicine, Washington University School of Medicine, St. Louis, Missouri, United States of America; 4 Shriners Hospitals for Children–St. Louis, St. Louis, Missouri, United States of America; University of Arizona, UNITED STATES

## Abstract

Chikungunya virus (CHIKV) is an arthritogenic alphavirus that acutely causes fever as well as severe joint and muscle pain. Chronic musculoskeletal pain persists in a substantial fraction of patients for months to years after the initial infection, yet we still have a poor understanding of what promotes chronic disease. While replicating virus has not been detected in joint-associated tissues of patients with persistent arthritis nor in various animal models at convalescent time points, viral RNA is detected months after acute infection. To identify the cells that might contribute to pathogenesis during this chronic phase, we developed a recombinant CHIKV that expresses Cre recombinase (CHIKV-3ʹ-Cre). CHIKV-3ʹ-Cre replicated in myoblasts and fibroblasts, and it induced arthritis during the acute phase in mice. Importantly, it also induced chronic disease, including persistent viral RNA and chronic myositis and synovitis similar to wild-type virus. CHIKV-3ʹ-Cre infection of tdTomato reporter mice resulted in a population of tdTomato^+^ cells that persisted for at least 112 days. Immunofluorescence and flow cytometric profiling revealed that these tdTomato^+^ cells predominantly were myofibers and dermal and muscle fibroblasts. Treatment with an antibody against Mxra8, a recently defined host receptor for CHIKV, reduced the number of tdTomato^+^ cells in the chronic phase and diminished the levels of chronic viral RNA, implicating these tdTomato^+^ cells as the reservoir of chronic viral RNA. Finally, isolation and flow cytometry-based sorting of the tdTomato^+^ fibroblasts from the skin and ankle and analysis for viral RNA revealed that the tdTomato^+^ cells harbor most of the persistent CHIKV RNA at chronic time points. Therefore, this CHIKV-3ʹ-Cre and tdTomato reporter mouse system identifies the cells that survive CHIKV infection *in vivo* and are enriched for persistent CHIKV RNA. This model represents a useful tool for studying CHIKV pathogenesis in the acute and chronic stages of disease.

## Author summary

Chikungunya virus (CHIKV) is spread by mosquitoes and causes severe joint and muscle pain. Approximately 30 to 60 percent of people infected with CHIKV continue to experience joint pain for months to years after the initial infection. However, the cause of this persistent joint pain is unclear, as replicating virus cannot be detected during the chronic phase. We have developed a reporter virus system to permanently mark cells infected by CHIKV. Using this system, we show in mice that marked cells surviving CHIKV infection are present for at least 112 days after initial virus inoculation. The marked cells are a mixture of muscle and skin cells. We also show that treatment of mice with an antibody that blocks receptor engagement and CHIKV infection reduces the number of marked cells in the muscle and skin. Finally, we have demonstrated that surviving tdTomato^+^ cells contain most of the persistent CHIKV RNA. This reporter virus system represents a useful tool for identifying and isolating cells that harbor chronic viral RNA in order to study chronic disease pathogenesis.

## Introduction

Chikungunya virus (CHIKV) is a globally re-emerging arthropod-transmitted virus that originally was identified in Tanzania in 1952 [1–3]. Up until the 2000s, CHIKV was considered a self-limiting virus of relatively minimal concern; however, within the last 15 years, CHIKV has reemerged with increased virulence and range. In 2004, an East/Central/South African (ECSA) lineage [4] of CHIKV caused an epidemic in Kenya with over 13,000 cases, the first large epidemic in decades [5]. By 2005, the virus spread to La Réunion Island off the east coast of Madagascar, where it infected over 200,000 people [6,7]. The La Réunion epidemic included the first reports of increased pathogenicity, including neurological symptoms, intrapartum transmission, and approximately 250 deaths [8–10]. The virus subsequently established endemic infection cycles in tropical regions including India and the South Pacific and also caused isolated outbreaks in Europe [11]. In 2013, an Asian lineage strain of CHIKV spread to the Americas and has caused nearly 2 million suspected cases in the Caribbean, Central America, and South America [11–14].

An estimated 75–95% of people who acquire CHIKV by a mosquito bite [15–17] experience an acute disease characterized by fever, rash, arthralgia, and myalgia that lasts for approximately one to two weeks [6,18,19]. Between 30% to 60% of CHIKV infected patients develop chronic joint and muscle pain that lasts for months to years after the acute infection [20–22]. Although CHIKV disease is rarely fatal, the acute and chronic arthritis cause substantial morbidity [20]. Furthermore, there are no approved vaccines or therapeutics for CHIKV, and over-the-counter medications provide little relief [23]. Despite its potential for causing morbidity, the mechanism of chronic CHIKV pathogenesis is poorly understood.

Chronic CHIKV disease is characterized by inflammation and immune system activation. A number of pro-inflammatory cytokines are elevated in serum during chronic CHIKV disease, including IL-1β, IL-6, and G-CSF [24–27]. CHIKV patients with chronic symptoms also have higher levels of activated CD8^+^ T cells and NK cells, resembling seronegative rheumatoid arthritis [28]. Multiple studies have attempted to identify predictive markers for CHIKV-induced chronic arthralgia [18,29–32]. While these studies agree that chronic CHIKV pathogenesis is characterized by a chronic inflammatory state, they do not define consistent biomarkers or disease mechanisms.

There is evidence that CHIKV antigen may persist chronically in tissues. CHIKV antigen was detected in synovial macrophages of one patient 18 months after infection [18], as well as in a human muscle specimen at least three months after acute infection [33]. Cells positive for CHIKV antigen also can be detected in persistently-infected macaques [34] or wild-type (WT) mice [35]. Recombinant CHIKV expressing firefly luciferase also produced detectable luminescence during the chronic stage of disease [36–38]. However, these and other studies have not demonstrated the presence of infectious virus in the chronic phase of disease [22,39].

One hypothesis for chronic CHIKV pathogenesis is that non-infectious CHIKV RNA remains in infected tissues, and its dsRNA intermediates act as proinflammatory pattern-associated molecular patterns (PAMPs) [40]. Chronic CHIKV RNA can consistently be detected by RT-qPCR in animal models, including the joints and spleen of mice and the spleen, lymph nodes, and liver of macaques [34,41]. Detection of chronic CHIKV RNA in human samples has been less consistent. One study reported the presence of CHIKV E1 and nsP2 RNAs in a human synovial fluid sample, yet others have failed to detect chronic CHIKV RNA in patient serum or synovial fluid [18,22].

The cells that harbor the CHIKV RNA during chronic disease are unknown. Immunohistochemical analysis infrequently detects CHIKV antigen-positive cells in WT mice, possibly due to the insensitivity of this technique [35]. Moreover, studies using RNA *in situ* hybridization (ISH) for detection of CHIKV RNA have not been published beyond acute time points in mice. Thus, to understand chronic CHIKV pathogenesis, there is a need to identify the cells that harbor viral RNA and to determine why the immune response cannot clear this persistent RNA.

To begin to address these questions, we established a model that permanently marks cells infected by CHIKV. We engineered a recombinant CHIKV infectious cDNA clone that encodes for Cre recombinase, as has been done previously for influenza [42,43] and herpes simplex viruses [44,45]. We demonstrate that this CHIKV-Cre virus infects and replicates in cell types targeted by CHIKV including myoblasts and fibroblasts and induces acute arthritis in mice. Importantly, this virus established chronic disease with persistence of viral RNA occurring for weeks after the acute infection. By combining this Cre expressing virus with reporter mice, we identified infection-marked cells in the chronic phase of disease and showed that these cells were enriched for persistent CHIKV RNA. This system provides a powerful tool to explore chronic CHIKV pathogenesis and the host determinants that contribute to disease.

## Results

### Generation and characterization of CHIKV expressing Cre recombinase

**Fig 1:**
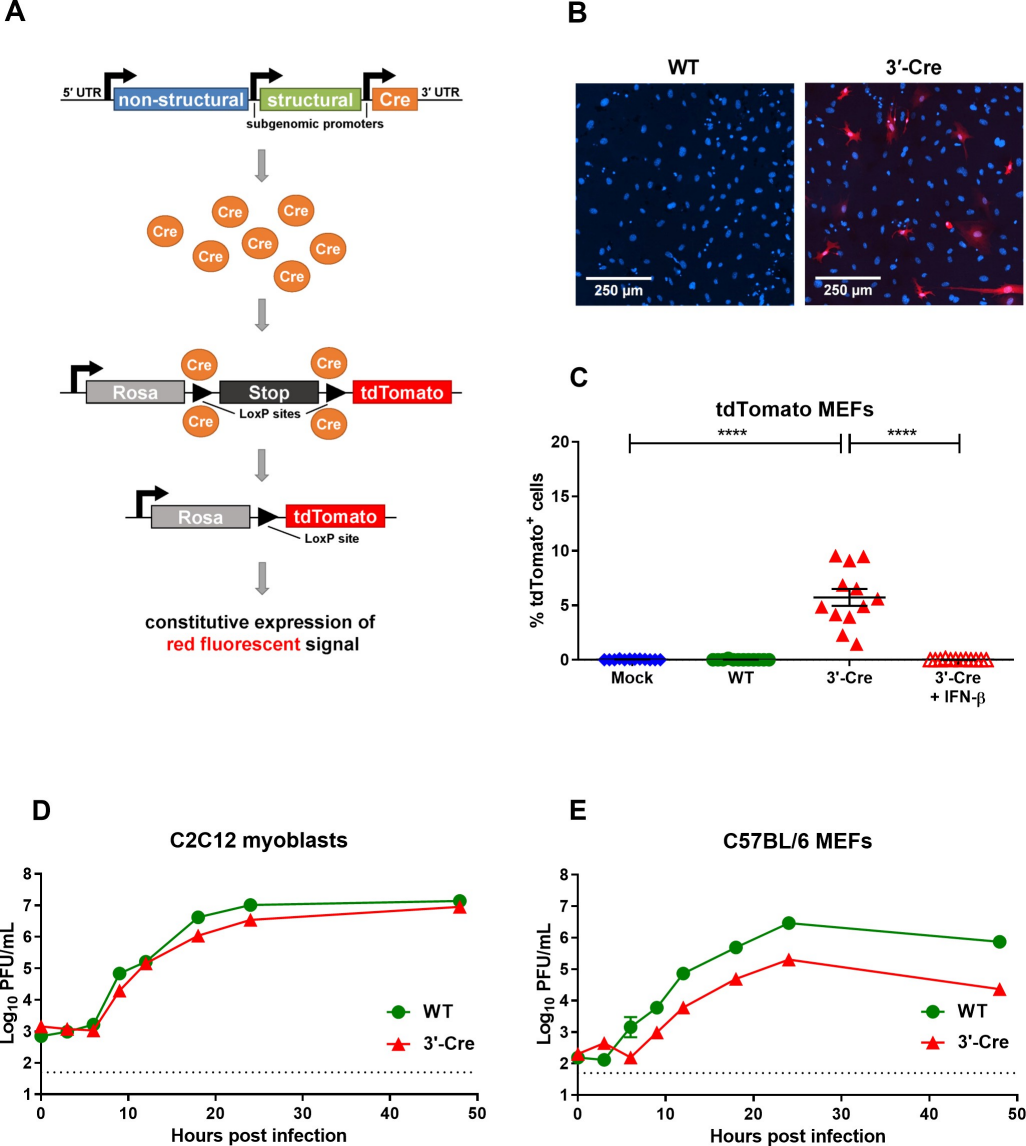
CHIKV-3ʹ-Cre marks reporter cells *in vitro* and productively infects muscle cells and fibroblasts. (A) Scheme of CHIKV-3ʹ-Cre and tdTomato reporter mouse system. (B-C) MEFs isolated from tdTomato reporter mice were analyzed 2 days after infection. (B) Representative images of CHIKV-WT or CHIKV-3ʹ-Cre infected tdTomato MEFs. Blue shows DAPI staining, and red is tdTomato. (C) The percentage of total DAPI^+^ cells that were tdTomato^+^ was quantified by confocal microscopy for tdTomato MEFs that were mock-infected (mock) or infected at an MOI of 10 with CHIKV-WT (WT), CHIKV-3ʹ-Cre (3ʹ Cre), or CHIKV-3ʹ-Cre pretreated with ~100 U IFN-β (3ʹ Cre + IFN-β), as described in the Methods. Representative growth curves of (D) C2C12 myoblasts or (E) C57BL/6 MEFs infected with CHIKV-WT (green circles) or CHIKV-3ʹ-Cre (red triangles) at an MOI of 1. Data for each condition in C were pooled from 2–3 independent experiments; data in C were analyzed with an ordinary one-way ANOVA with Tukey’s post-test. All error bars indicate mean with standard error of the mean (SEM). (*, *P* < 0.05; **, *P* < 0.01; ***, *P* < 0.001; ****, *P* < 0.0001).

To identify cells surviving CHIKV infection, we generated a recombinant CHIKV using the La Réunion infectious cDNA clone, LR2006 OPY1, (denoted CHIKV-WT) and engineered it to express the bacteriophage Cre recombinase gene under control of a second subgenomic promoter. Two versions of the Cre recombinase virus were generated: in one version, the second subgenomic promoter and Cre recombinase was introduced between the non-structural and structural genes (denoted CHIKV-5ʹ-Cre); in the second version, the subgenomic promoter and Cre recombinase were inserted downstream of the structural genes (denoted CHIKV-3ʹ-Cre) (**S1A Fig**). The infection of tdTomato reporter mice with our recombinant viruses results in the expression of Cre recombinase, excision of the stop cassette within the reporter construct, and constitutive expression of the fluorescent tdTomato protein. Thus, the fluorescent reporter protein will be expressed in all cells where Cre recombinase was expressed, even when viral replication is abrogated (**Fig 1A**). This system will help determine whether individual cells can survive the initial CHIKV infection.

We first tested the ability of CHIKV-3ʹ-Cre to function in cell culture. Murine embryonic fibroblasts (MEFs) from tdTomato reporter mice were isolated and inoculated with either CHIKV-WT or CHIKV-3ʹ-Cre. Two days post infection (dpi), a population of tdTomato^+^ cells was detected following CHIKV-3ʹ-Cre infection, but not following mock treatment or infection with CHIKV-WT (**Fig 1B and 1C**). Infection with CHIKV-5ʹ-Cre also induced reporter expression, although in a lower number of cells than CHIKV-3ʹ-Cre, and this difference was seen across several multiplicities of infection (MOI) (**S1B Fig**). To determine if viral replication was required to activate the reporter, we pretreated cells with IFN-β, which inhibits alphavirus replication [46,47]. Pretreatment with IFN-β prevented expression of the tdTomato reporter by cells infected with the CHIKV-3ʹ-Cre virus (**Fig 1C**). Similar results were observed with CHIKV-5ʹ-Cre (**S1C Fig**). Taken together, these results suggest that viral replication and expression of Cre recombinase in the infected cell is necessary for activation of tdTomato expression.

We next assessed the ability of CHIKV-3ʹ-Cre to replicate in muscle cells and fibroblasts, two cell types that are major targets of CHIKV infection [18,19,46–50]. C2C12 myoblasts or MEFs from WT C57BL/6 mice were inoculated with CHIKV-WT or CHIKV-3ʹ-Cre at an MOI of 1, and viral replication was assessed at various time points. In C2C12 myoblasts, CHIKV-3ʹ-Cre replicated similarly to CHIKV-WT, though with slight attenuation at some time points (**Fig 1D**). CHIKV-3ʹ-Cre also replicated in MEFs, although unexpectedly it was more attenuated than CHIKV-WT in this cell (**Fig 1E**). Similar results were seen when C2C12 cells or MEFs were infected at an MOI of 0.05 and with CHIKV-5ʹ-Cre (**S1D and S1E Fig**). Therefore, the CHIKV-3ʹ-Cre virus retains its ability to replicate in key target cells during infection, although with variable levels of attenuation in myoblasts and fibroblasts.

### CHIKV-3ʹ-Cre retains its pathogenic properties to induce acute arthritis

**Fig 2:**
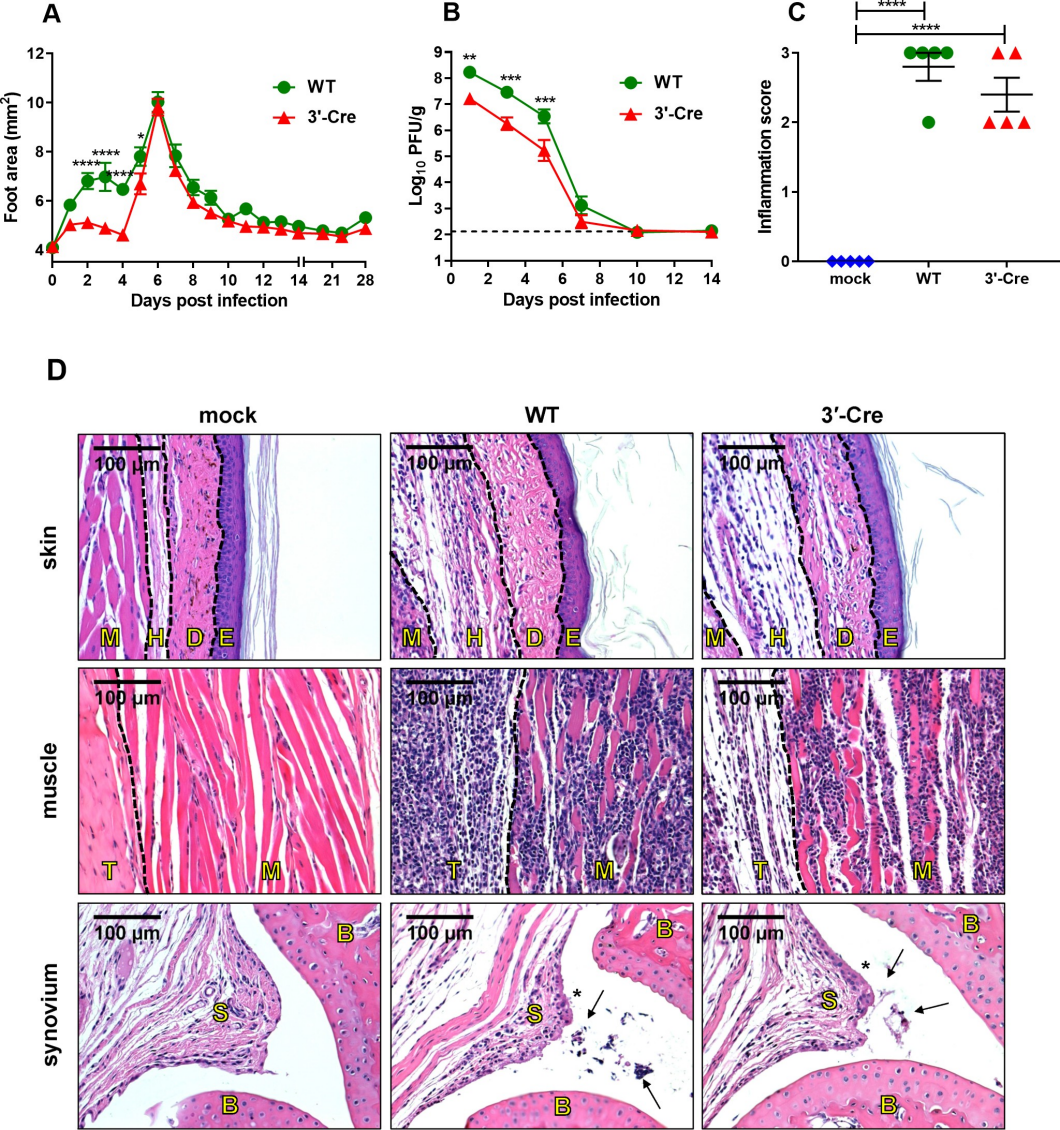
CHIKV-3ʹ-Cre retains its pathogenic properties to induce acute arthritis. (A) Swelling of the ipsilateral feet of mice inoculated with 10^6^ PFU of CHIKV-WT (green circles) or CHIKV-3ʹ-Cre (red triangles). Data were pooled from two independent experiments with n = 10 for each virus. (B) Levels of infectious virus in the ipsilateral ankle during acute infection as measured by plaque assay, normalized to gram of tissue. Each time point for each virus represents 5–7 mice and were pooled from 2–4 independent experiments. The dashed line represents the limit of detection. (C-D) Mice were mock-infected (mock, blue diamonds) or infected with 10^6^ PFU CHIKV-WT (WT, green circles) or CHIKV-3ʹ-Cre (3ʹ-Cre, red triangles), and ipsilateral ankles at 7 dpi were analyzed histologically after hematoxylin and eosin staining of sections. (C) Ankles were scored for histological damage as described in the Methods. (D) Representative images of the skin, muscle, and synovium. The skin and associated tissue are divided (from left to right) into muscle (M), hypodermis (H), dermis (D), and epidermis (E). The muscle sections are divided into tendon (T) and muscle (M). The synovium sections show synovium (S) and bone (B), with asterisks indicating synovial inflammation and arrows indicating immune infiltrates into the synovial cavity. Samples were pooled from two independent experiments with five mice for each condition. Data in A were analyzed with a two-way repeated measures (RM) ANOVA with Bonferroni’s post-test. Data in B were log-transformed and then analyzed with an ordinary two-way ANOVA with Bonferroni’s post-test. Data in C were analyzed with an ordinary one-way ANOVA with Tukey’s post-test. All error bars indicate SEM. (*, *P* < 0.05; **, *P* < 0.01; ***, *P* < 0.001; ****, *P* < 0.0001).

A mouse model of CHIKV infection has been shown to recapitulate several aspects of human infection [50–52]. Acute infection is characterized by foot swelling that resolves within 10–14 dpi and replication and dissemination of the virus to joint-associated tissues, muscle, spleen, and blood. In addition, edema and immune cell infiltration into the joint and muscle are observed. We therefore evaluated the ability of CHIKV-3ʹ-Cre to induce acute clinical disease. C57BL/6 mice were inoculated with either CHIKV-WT or CHIKV-3ʹ-Cre into the foot and analyzed for swelling, viral replication, and histopathology. Injection of each virus resulted in a biphasic pattern of acute ipsilateral foot swelling with peaks seen at 3 and 6 dpi and resolution by 10–14 dpi (**Fig 2A**). Although the initial swelling peak at 2–3 dpi, thought to be caused by edema and monocyte infiltration [53], was reduced during CHIKV-3ʹ-Cre infection, the latter and more pronounced swelling peak at 6 dpi, which is driven by infiltration of monocytes and CD4^+^ T cells [37,53], was equivalent between the two viruses (**Fig 2A**). Similar results were seen with CHIKV-5ʹ-Cre (**S2A Fig**).

We next measured viral titers in the ipsilateral ankle of infected mice. High viral loads were detected in both the CHIKV-WT and CHIKV-3ʹ-Cre infected mice at 1, 3, and 5 dpi, although CHIKV-3ʹ-Cre was mildly attenuated with ~10-fold lower levels than CHIKV-WT. Infectious virus from either CHIKV-WT or CHIKV-3ʹ-Cre was undetectable by 10–14 dpi (**Fig 2B**). Again, similar results were seen with CHIKV-5ʹ-Cre (**S2B Fig**). Infection with CHIKV-WT also resulted in viremia and replication at distal sites including the ipsilateral quadriceps muscle, contralateral ankle, and spleen. Whereas viral replication of both CHIKV-3ʹ-Cre and CHIKV-5ʹ-Cre was detected in these distal tissues, it was attenuated compared to CHIKV-WT (**S2C–S2F Fig**).

We also utilized RNA ISH to detect viral RNA in selected tissue compartments. RNA ISH was performed on the ipsilateral foot and ankle at 2 dpi with CHIKV-WT or CHIKV-3ʹ-Cre. CHIKV E1 RNA was detected for both viruses in skeletal muscle, skin, and synovium (**S3A Fig**). CHIKV-3ʹ-Cre had a similar tissue tropism to CHIKV-WT, although staining for CHIKV-3ʹ-Cre was less intense than with CHIKV-WT. Notwithstanding these differences, CHIKV-3ʹ-Cre had a similar overall tissue tropism to CHIKV-WT.

CHIKV infection results in moderate to severe arthritis that can be detected at acute and subacute time points in animal models [39,41]. This is characterized by edema and swelling within the skin and subcutaneous tissue and cellular infiltrates in the joint space, muscle, and tenosynovial tissues. Specimens comprising the ipsilateral foot and ankle were harvested seven days after mock infection or infection with CHIKV-WT or CHIKV-3ʹ-Cre, and tissue sections were evaluated for inflammation and acute arthritis (**Fig 2C and 2D**). Histopathological examination revealed substantial edema, especially in the dermis and hypodermis and inflammation with tissue damage in the footpad, skeletal muscle, and tenosynovial joint tissues in mice infected by either virus compared to mock-infected animals (**Fig 2D**). Based on histological analysis, the overall severity of the inflammatory response in the joint space, muscle, and tenosynovial soft tissues was indistinguishable between CHIKV-WT and CHIKV-3ʹ-Cre at 7 dpi (**Fig 2C**). Similar results were seen with CHIKV-5ʹ-Cre at 7 dpi (**S3B and S3C Fig**). Thus, CHIKV-3ʹ-Cre retains the pathogenic potential to induce acute musculoskeletal disease.

### CHIKV-3ʹ-Cre retains its pathogenic properties to induce chronic disease

Chronic CHIKV arthritis is characterized by persistent myositis and chronic synovial inflammation. In addition, persistent viral RNA can be detected in musculoskeletal tissues in mice long after acute disease, despite the absence of detectable replicating virus [39,41]. Therefore, we next evaluated if the CHIKV-3ʹ-Cre virus could establish chronic disease.

**Fig 3:**
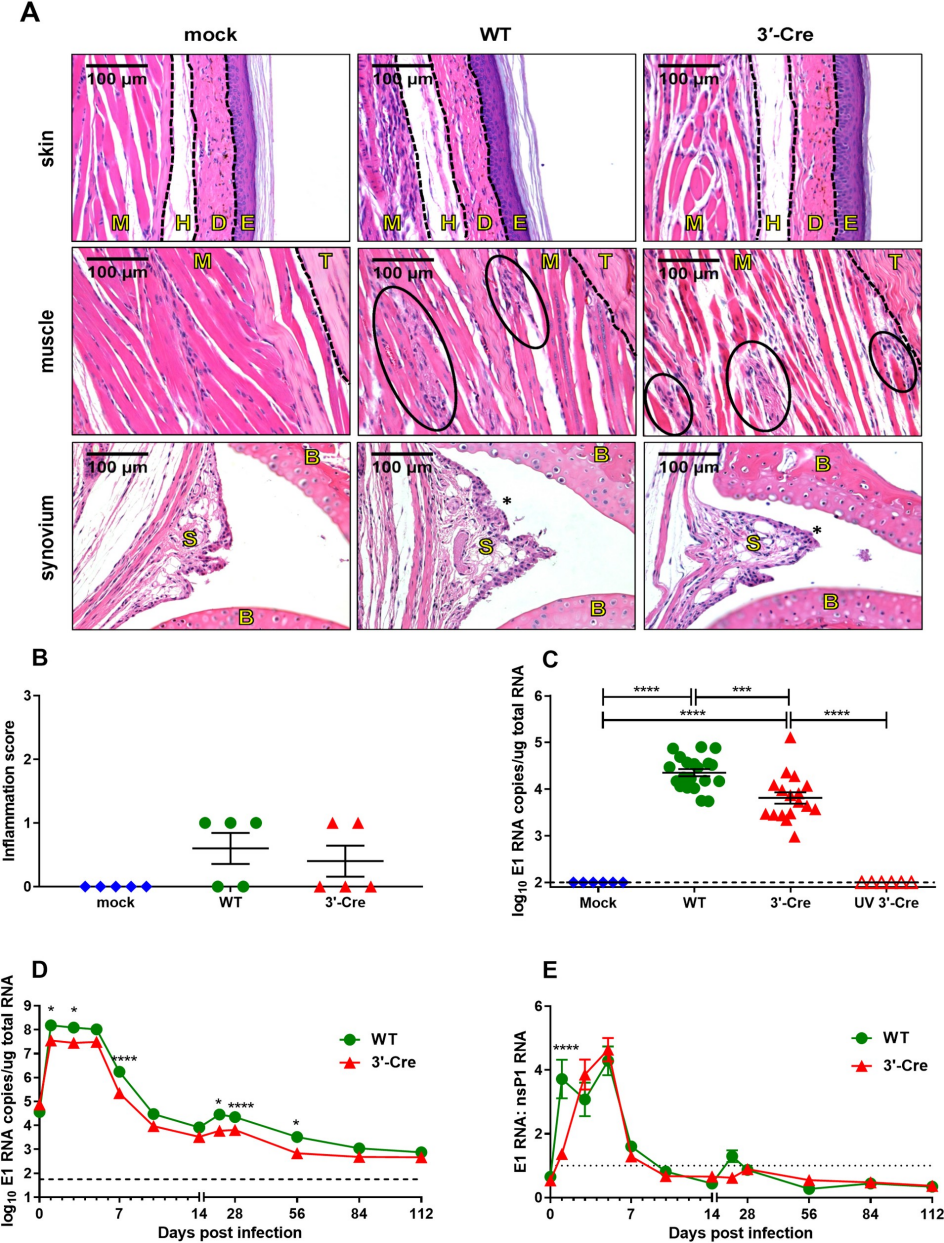
CHIKV-3ʹ-Cre retains its pathogenic properties to induce chronic disease. (A-B) Mice were mock-infected (mock, blue diamonds) or inoculated with 10^6^ PFU CHIKV-WT (WT, green circles) or CHIKV-3ʹ-Cre (3ʹ-Cre, red triangles), and ipsilateral ankles were taken for H&E histology at 28 dpi. (A) Representative images of the skin, muscle, and synovium. Black ovals mark focal patches of cellular filtrates. The synovium sections show synovium (S) and bone (B), with asterisks indicating synovial inflammation and proliferation. (B) Ankles were scored for histological damage as described in Methods. Samples were pooled from two independent experiments with five mice for each condition. (C-E) Mice were mock-infected (mock, blue diamonds) or inoculated with 10^6^ PFU of CHIKV-WT (WT, green circles), CHIKV-3ʹ-Cre (3ʹ Cre, red triangles) or UV-inactivated CHIKV-3ʹ-Cre (UV 3ʹ-Cre, open triangles), and at the indicated times post infection RNA was isolated from the ipsilateral ankles and viral E1 or nsP1 RNA copy number was measured by RT-qPCR. RNA levels were normalized to total μg of RNA isolated for each sample. (C) Viral E1 RNA levels in the ipsilateral ankles were assayed at 28 dpi. Data were pooled from 3 independent experiments. (D) Viral E1 RNA levels were measured in the ipsilateral ankles of the indicated mice at time points ranging from 0 to 112 dpi. The dashed line for C and D represents limit of detection. (E) The ratio of viral E1 RNA to viral nsP1 RNA in the same samples as D. The dotted line represents an E1:nsP1 ratio of 1. For D-E, each time point for each virus represents 5–20 mice and was pooled from 2–4 independent experiments. Data in C and D were log-transformed prior to analysis. Data in B and C were each analyzed with an ordinary one-way ANOVA using Tukey’s post-test, with only relevant comparisons shown. Data in D and E were analyzed with an ordinary two-way ANOVA using Bonferroni’s post-test. All error bars indicate SEM. (*, *P* < 0.05; **, *P* < 0.01; ***, *P* < 0.001; ****, *P* < 0.0001).

Ipsilateral foot and ankle joints were harvested twenty-eight days after inoculation with media (mock infection), CHIKV-WT, or CHIKV-3ʹ-Cre. Compared to specimens harvested at 7 dpi (**Fig 2C and 2D**), there was markedly less inflammation and tissue damage in all groups at 28 dpi (**Fig 3A and 3B**), consistent with the comparatively mild histological findings previously described in CHIKV-associated chronic arthritis in mice [39,41]. Histological examination showed patchy areas of chronic inflammatory infiltrates within the muscle, with some synovial proliferation within the joints, consistent with resolving arthritis in both the CHIKV-WT and CHIKV-3ʹ-Cre infected mice, but not in mock-infected mice (**Fig 3A**). The overall severity of the inflammation in the joint space, muscle, and tenosynovial soft tissues was indistinguishable between CHIKV-WT and CHIKV-3ʹ-Cre at 28 dpi (**Fig 3B**). Similar results were observed for CHIKV-5ʹ-Cre (**S4A and S4B Fig**). Thus, CHIKV-3ʹ-Cre establishes chronic CHIKV arthritis.

We next evaluated if viral RNA persisted in tissues after infection with the CHIKV-3ʹ-Cre virus. CHIKV viral E1 RNA was detected by RT-qPCR in the ipsilateral ankles of mice infected with either CHIKV-WT or CHIKV-3ʹ-Cre at 28 dpi, although at this time point the level of viral RNA was slightly decreased (~3.5-fold) in the CHIKV-3ʹ-Cre infected mice compared to CHIKV-WT (**Fig 3C**). As expected, mock infection or infection with UV-inactivated CHIKV-3ʹ-Cre did not result in a positive signal for viral RNA at chronic phase time points (**Fig 3C**). Persistent levels of chronic viral RNA were detected through 112 dpi (16 weeks) following infection with either virus, and both viruses established similar levels of viral RNA at late time points (**Fig 3D**). Again, similar results were observed for CHIKV-5ʹ-Cre (**S4C Fig**).

At peak productive infection, engagement of the subgenomic promoter produces a subgenomic RNA segment encoding for the structural proteins [54–56]. As such, this results in more copies of structural proteins, such as E1, being produced than non-structural genes, such as nsP1, during active RNA replication. Consistent with this description and as seen previously [41], during the first 7 days of infection, the ratio of E1 to nsP1 RNA copies was greater than 1 for both CHIKV-WT and CHIKV-3ʹ-Cre indicating active viral replication was occurring (**Fig 3E**). However, after 7 dpi the ratio of E1 to nsP1 was approximately 1, and the viral RNA levels decreased with a similar time course for both viruses. Similar results were observed for CHIKV-5ʹ-Cre (**S4D Fig**). Thus, the subgenomic promoter likely is active only during the first week of acute infection, which is supported by infectious virus only being detected during this period (**Fig 2B**).

### CHIKV-3ʹ-Cre identifies dermal and muscle fibroblasts and myofibers as target cells during chronic infection

**Fig 4:**
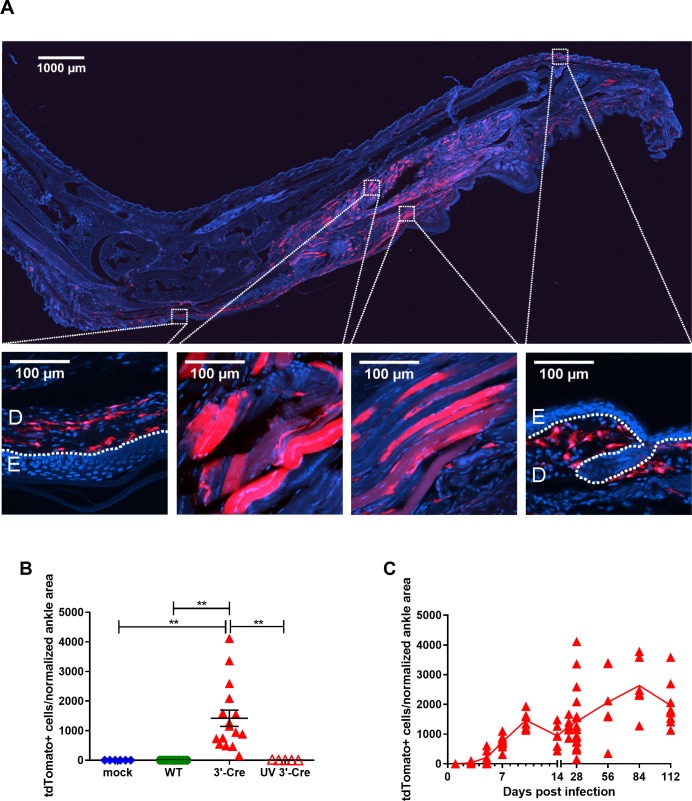
Cells are marked by CHIKV-3ʹ-Cre at time points in the chronic phase. tdTomato mice were mock-infected (mock, blue circles) or inoculated with 10^6^ PFU CHIKV-WT (WT, green circles), CHIKV-3ʹ-Cre (3ʹ-Cre, red triangles), or UV-inactivated CHIKV-3ʹ-Cre (UV 3ʹ-Cre, open red triangles), and ipsilateral feet and ankles were processed at 28 dpi. (A) Representative image of frozen sections of whole ankle and foot 28 dpi with CHIKV-3ʹ-Cre. Blue shows DAPI staining, and red is tdTomato; scale bar represents 1000 μm. Higher magnification inset images of skin and muscle from mice infected with CHIKV-3ʹ-Cre. Scale bars represent 100 μm; the skin is divided into dermis (D) and epidermis (E). (B) The total number of tdTomato^+^ cells was quantified at 28 dpi and normalized to ankle area as described in the Methods. (C) Time course of tdTomato mice infected with 3ʹ-Cre from 1 to 112 dpi. Data from B and C were pooled from 2–4 independent experiments. B was analyzed with an ordinary one-way ANOVA using Sidak’s post-test. All error bars indicate SEM. (*, *P* < 0.05; **, *P* < 0.01; ***, *P* < 0.001; ****, *P* < 0.0001).

Previous reports have indicated that CHIKV is highly cytopathic and induces cell death in the majority of cells that it infects [57,58]. To test whether cells could survive CHIKV infection *in vivo*, tdTomato reporter mice were inoculated with media (mock), CHIKV-WT, or CHIKV-3ʹ-Cre and analyzed for the presence of tdTomato^+^ cells at chronic phase time points. Notably, tdTomato^+^ cells were observed in the ipsilateral foot 28 dpi with CHIKV-3ʹ-Cre, but not in WT C57BL/6 or reporter mice that were mock-infected, infected with CHIKV-WT, or infected with UV-inactivated CHIKV-3ʹ-Cre (**Fig 4A and 4B**, **S5A Fig**). Infection with CHIKV-5ʹ-Cre infection also resulted in tdTomato^+^ cells in the ipsilateral foot, but at a much lower level than CHIKV-3ʹ-Cre (**S5B–S5D Fig**). TdTomato^+^ cells were detected up to at least 112 days (16 weeks) after infection with CHIKV-3ʹ-Cre (**Fig 4C**).

Closer examination of ipsilateral feet infected with CHIKV-3ʹ-Cre revealed that the majority of tdTomato^+^ cells were concentrated in skeletal muscle and the dermal layer of the skin (**Fig 4A**). In the muscle, the tdTomato^+^ cells appeared to be a mixture of long, multi-nucleated myofibers and small, uninucleated non-myofiber cells (**Fig 4A**). A high number of uninuclear tdTomato^+^ cells also were observed in the dermis of the skin. Other connective tissues such as bone, synovium, and tendons contained rare populations of tdTomato^+^ cells (**S5E Fig**).

**Fig 5:**
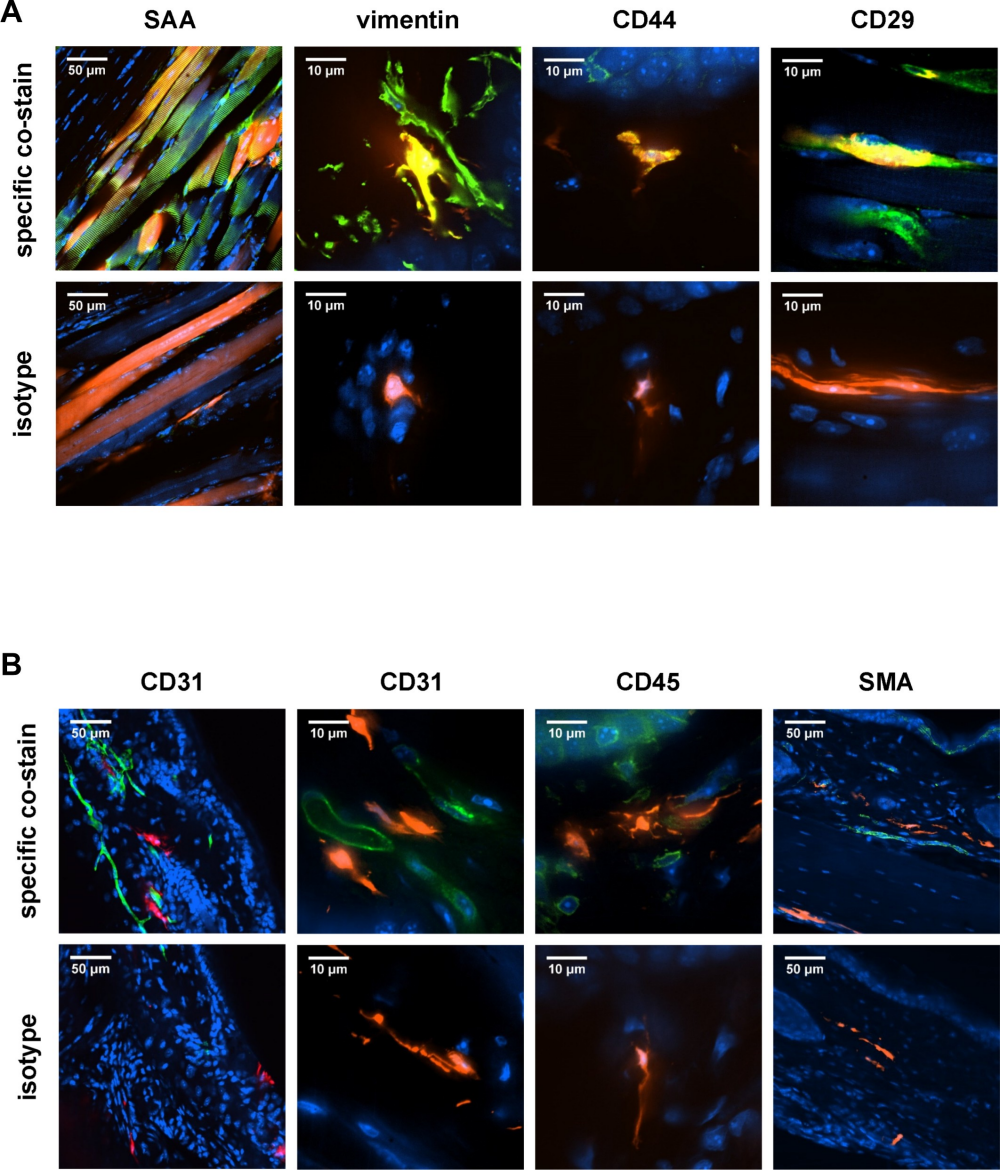
tdTomato^+^ cells colocalize with markers for myofibers and fibroblasts. tdTomato mice were infected with 10^6^ PFU CHIKV-3ʹ-Cre (3ʹ-Cre), and ipsilateral ankles were processed at 28 dpi. (A) Frozen sections were stained for markers of myofibers (SAA) or fibroblasts (vimentin, CD44, or CD29), with the corresponding isotype control shown below each co-stain. (B) Frozen sections were stained for endothelial cell (CD31), hematopoietic cell (CD45), or smooth muscle cell (SMA) markers, with the corresponding isotype control shown below each co-stain. Blue shows DAPI staining, red is tdTomato, and green is the indicated co-staining marker; scale bars represent 10 or 50 μm as indicated. Images are representative of 2–3 experiments with 3–8 mice reviewed for each co-staining.

To define the tdTomato^+^ cells, we performed co-staining studies. Since the majority of the tdTomato^+^ cells were localized to muscle and the dermis, we focused our analysis on these two areas. In the muscle, the long multi-nucleated, striated cells that histologically were consistent with skeletal muscle fibers co-stained with sarcomeric alpha actinin (SAA) (**Fig 5A**), confirming that the tdTomato^+^ cells were skeletal muscle cells. The small, uninucleated tdTomato^+^ cells in the muscle were negative for SAA staining.

CHIKV is known to infect fibroblasts both *in vitro* and *in vivo* [46,47], and the morphology of some tdTomato^+^ cells in muscle and skin was consistent with being fibroblasts. Although there is no specific marker for fibroblasts, tdTomato^+^ cells in the muscle co-stained with vimentin, an intermediate filament that is expressed highly in fibroblasts (**Fig 5A**). In the dermis of the skin, many of the tdTomato^+^ cells also co-stained strongly with vimentin (**Fig 5A**). Some of the tdTomato^+^ cells in the skin or muscle also co-stained with CD44 and CD29, additional fibroblast markers (**Fig 5A**). As these mesenchymal markers can be expressed in other cell types, sections also were stained with CD31 (endothelial cell marker), CD45 (hematopoietic cell marker), or smooth muscle actin (SMA, myofibroblast and smooth muscle marker). TdTomato^+^ cells in the skin and muscle did not co-stain with any of these markers (**Fig 5B)**.

**Fig 6:**
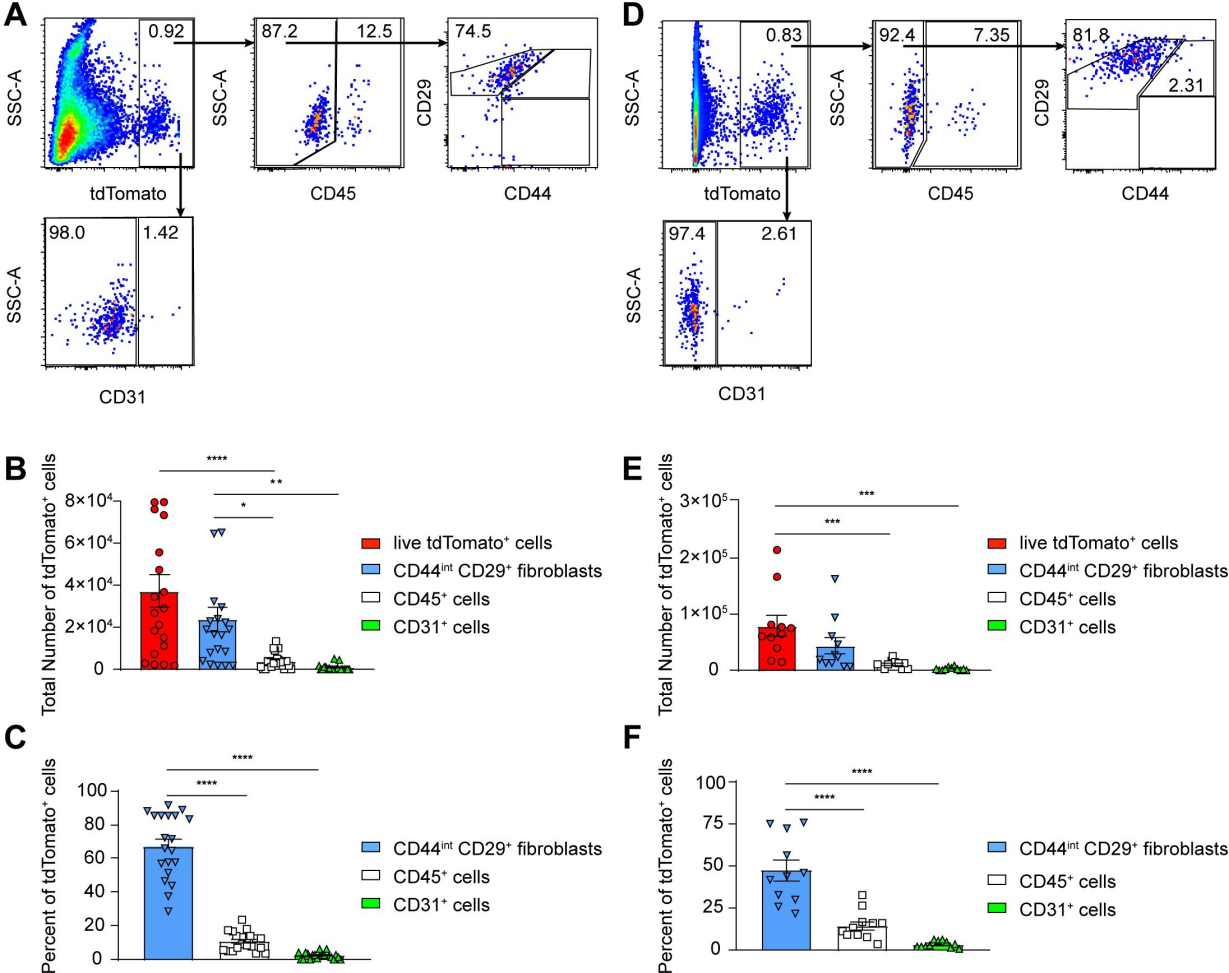
tdTomato^+^ uninuclear cells isolated from the skin and ankle are predominantly CD29^+^ CD44^int^ fibroblasts. tdTomato mice were infected with 10^5^ PFU CHIKV-3ʹ-Cre (3ʹ-Cre) and at 28 dpi the skin (A-C) or ankle tissue (D-F) from the ipsilateral foot/ankle were processed and analyzed by flow cytometry. (A and D) Representative flow cytometry plots showing the tdTomato^+^ population of cells isolated from the (A) skin and (D) ankle and co-staining with CD45, CD29, CD44, and CD31. (B and E) Quantification of the total number of live tdTomato^+^ cells isolated from the (B) skin and (E) ankle and co-staining with CD45, CD29/CD44, or CD31. (C and F) Percentage of tdTomato+ cells isolated from the (C) skin or (F) ankle that co-stain with CD45, CD29/CD44, or CD31. Data represents three (D-F) or four (A-C) independent experiments. Data were analyzed using an ordinary one-way ANOVA with Tukey’s post-test. All error bars indicate SEM. (*, *P* < 0.05; **, *P* < 0.01; ***, *P* < 0.001; ****, *P* < 0.0001).

We used flow cytometry to confirm the findings seen by microscopy. Since the muscle fibers made up a significant population of the tdTomato^+^ cells in the ankle tissue, and these cells could not be isolated and analyzed by flow cytometry, we initially characterized the tdTomato^+^ cells in the skin. tdTomato^+^ cells were isolated from the skin taken from the ipsilateral foot of infected reporter mice as described in the Methods. We then determined the number of tdTomato^+^ live leukocytes (CD45^+^), endothelial cells (CD31^+^), and fibroblasts (CD45^-^ CD29^+^ CD44^int^) (gating strategy in **S6A and S6B Fig)**. We isolated an average of 4x10^4^ tdTomato^+^ cells from the digested skin (**Fig 6B**), which constituted less than 1% of the total cells isolated. Of these tdTomato^+^ cells, 70–80% of them stained positively for markers associated with fibroblasts including CD29 and CD44 (**Fig 6B and 6C**). We observed that less than 5% of the tdTomato^+^ cells were CD31^+^ endothelial cells (**Fig 6B and 6C**). Although we did not detect co-staining of CD45 and tdTomato using immunofluorescence, we did detect a small population of CD45^+^ tdTomato^+^ cells in the skin, which consisted of less than 15% of the total tdTomato^+^ cells isolated (**Fig 6B and 6C**). However, the tdTomato mean florescence intensity of the CD45^+^ tdTomato^+^ cells was about 3-fold lower than the levels seen in the CD45^-^ tdTomato^+^ cells (**S6B and S6C Fig**).

Next, we examined the uninuclear cells in the ankle tissue using similar techniques (**Fig 6D,** complete gating strategy in **S6D Fig**). We observed approximately 8x10^4^ tdTomato^+^ cells in the ankle tissue, a two-fold increase in the total number of tdTomato^+^ cells compared to the skin (**Fig 6E**), which constituted less than 1% of the total uninuclear cells in the ankle tissue. Once again the majority of the tdTomato^+^ cells in the ankle tissue stained positively for CD29 and CD44 (approximately 50–60%), consistent with these cells being fibroblasts (**Fig 6E and 6F**). We observed that less than 5% of the tdTomato^+^ cells were CD31^+^ endothelial cells (**Fig 6E and 6F**), and less than 15% of the tdTomato^+^ cells stained positively with CD45, once again displaying a lower MFI of tdTomato staining than the CD45^-^ cells (**Fig 6F, S6E and S6F Fig**). Collectively, the immunofluorescence and the flow cytometric analyses suggest that the majority of the tdTomato^+^ cells are myofibers and dermal or muscle fibroblasts.

### Mice treated with anti-Mxra8 antibodies exhibit reduced numbers of tdTomato^+^ cells and levels of chronic viral RNA at chronic phase time points

**Fig 7:**
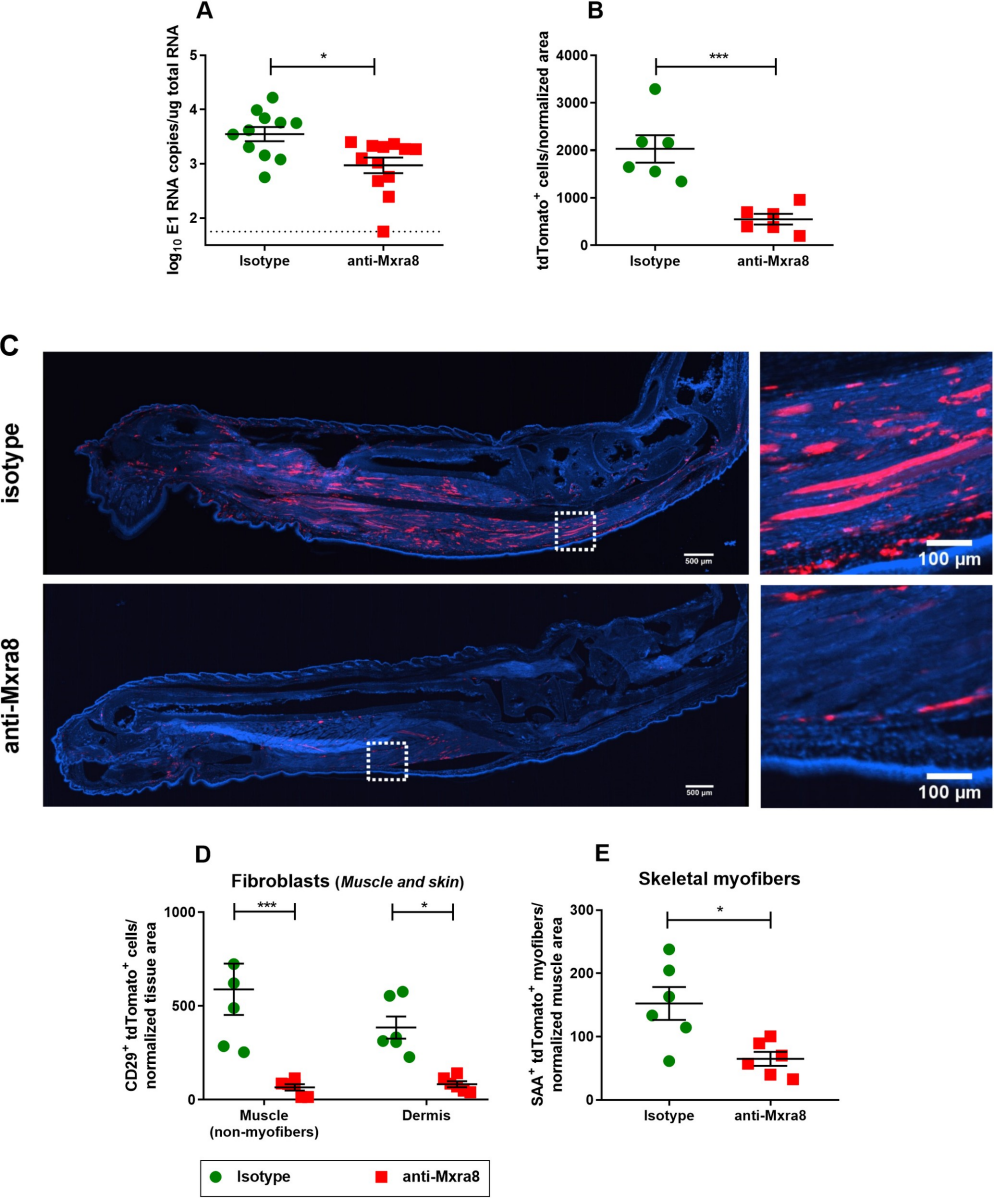
Anti-Mxra8 antibodies reduce levels of viral RNA and the number of tdTomato^+^ cells in the chronic phase of CHIKV infection. tdTomato mice were treated with anti-Mxra8 mAbs (red squares) or an isotype control antibody (green circles) as described in the Methods, inoculated with 10^6^ PFU CHIKV-3ʹ-Cre (3ʹ-Cre), and at 28 dpi mice were harvested for quantification of viral RNA and histological analysis. (A) Quantification of CHIKV E1 RNA in the ipsilateral ankle/foot of isotype or anti-Mxra8 treated mice. Data was pooled from three independent experiments. (B-F) Ipsilateral ankles/feet were harvested for histological analysis; data was pooled from two independent experiments. (B) Quantification of the total number of tdTomato^+^ cells in the ipsilateral ankles/feet. (C) Representative images of ipsilateral feet/ankles, with higher magnifications of dotted squares inset to the right. Blue shows DAPI staining, and red is tdTomato; scale bars represent 100 or 500 μm as indicated. (D-E) tdTomato^+^ cells were quantified by morphology and co-staining as described in the Methods: (D) CD29^+^ muscle and skin cells, and (E) SAA^+^ myofibers. Data in A were analyzed with a Mann-Whitney test. Data in B and E were analyzed with an unpaired t test. Data in D was analyzed with a two-way ANOVA using Sidak’s post-test. All error bars indicate SEM. (*, *P* < 0.05; **, *P* < 0.01; ***, *P* < 0.001; ****, *P* < 0.0001).

To assess if there is a correlation between the number tdTomato^+^ cells and persistent CHIKV RNA levels, we utilized anti-Mxra8 monoclonal antibodies (mAbs) to block a recently reported entry receptor for CHIKV, in combination with our reporter virus system. It was previously reported that treatment with anti-Mxra8 mAbs reduced CHIKV infection and foot swelling at 3 dpi during the acute phase of disease [59]. To assess the impact of anti-Mxra8 blockade on chronic infection, tdTomato reporter mice were treated with either isotype control or anti-Mxra8 mAbs 12 hours prior to infection and then at 4, 8, 12, 16, 20, and 24 dpi. Mice treated with anti-Mxra8 mAbs showed a significant reduction (73% decrease) in persistent viral RNA detected in the ipsilateral ankle 28 dpi compared to the isotype control mAb treated mice (**Fig 7A**).

Consistent with this data, the anti-Mxra8 treated mice had reduced numbers (75% decrease) of tdTomato^+^ cells in the ipsilateral ankle 28 days after infection as compared to the isotype control (**Fig 7B and 7C**). Quantification of cell co-staining demonstrated that the most significant reductions with anti-Mxra8 treatment were observed in tdTomato^+^ CD29^+^ fibroblasts in the muscle and skin (**Fig 7D**), with an 89% and 78% decrease, respectively. Anti-Mxra8 mAb treatment also reduced the number of tdTomato^+^ SAA^+^ myofibers by 57%, thus to a lesser extent than fibroblasts (**Fig 7E**). Overall, anti-Mxra8 mAb treatment reduced the amount of persistent viral RNA and altered the overall numbers and distribution of tdTomato^+^ cells in musculoskeletal tissues during the chronic phase of disease. These findings demonstrate a positive correlation between persistent CHIKV RNA levels and the number of tdTomato^+^ cells suggesting that these cells may harbor chronic RNA.

### tdTomato^+^ cells are enriched for persistent CHIKV RNA

**Fig 8:**
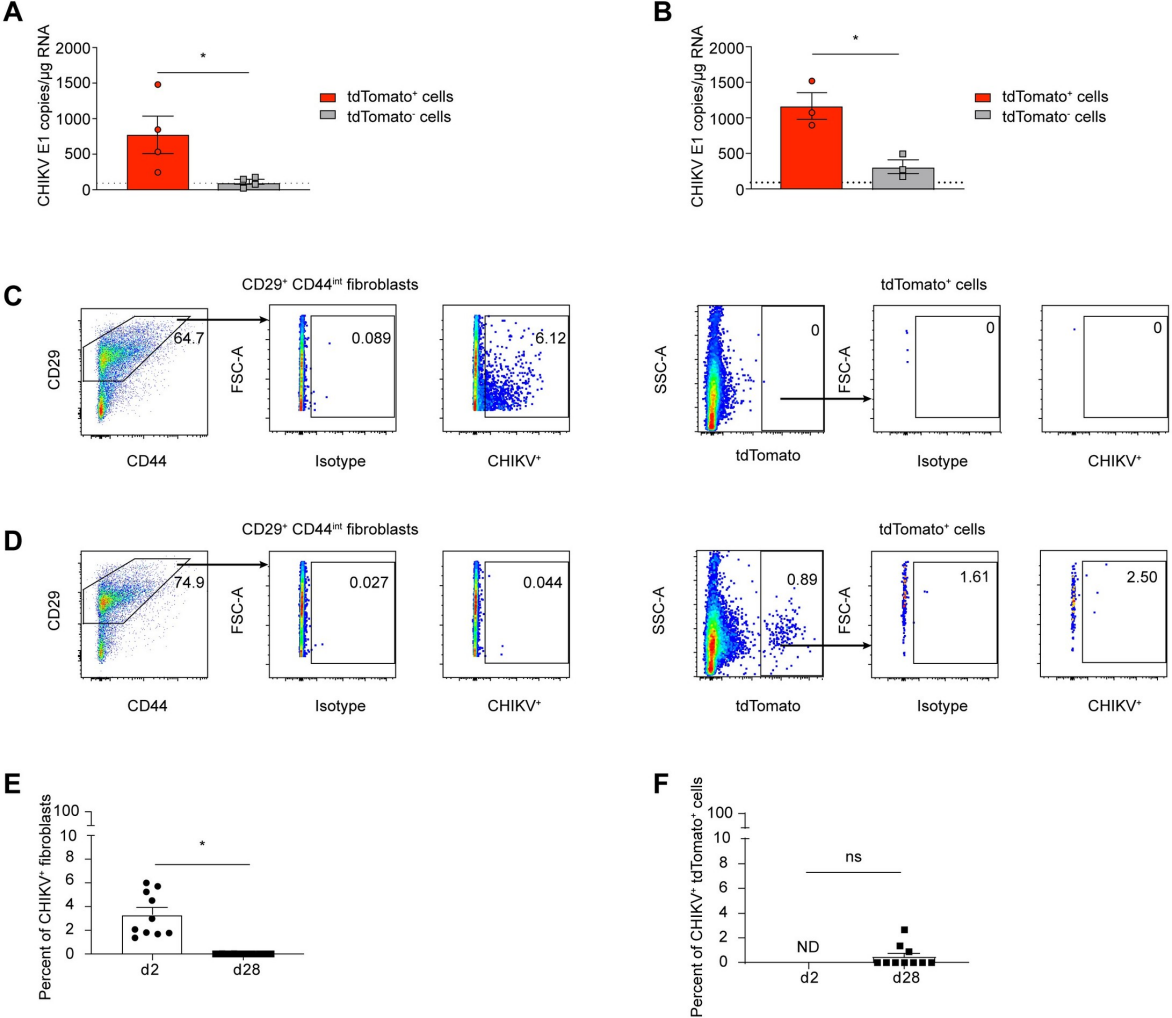
tdTomato^+^ cells harbor persistent CHIKV RNA but do not express CHIKV E1 or E2 proteins on their cell surface. tdTomato mice were infected with 10^5^ PFU of CHIKV-3ʹ-Cre (3ʹ-Cre), and skin or ankle was isolated from the ipsilateral foot at either 2 or 28 dpi and processed to generate single cell suspensions. (A-B) The CD45^-^ cells isolated from the skin (A) or ankle (B) were sorted into tdTomato^+^ and tdTomato^-^ populations and RNA was extracted, and analyzed for CHIKV E1 RNA by RT-qPCR. Each data point represents 5 pooled mice. (C, D) Representative flow plots of CD45^-^ CD29^+^ CD44^int^ fibroblasts (left panels) and tdTomato^+^ cells (right panels) staining with isotype control or anti-CHIKV Ab at 2 dpi (C) or 28 dpi (D). (E, F) Quantification of the percent of CHIKV^+^ fibroblasts (E) and CHIKV^+^ tdTomato^+^ cells (F) normalized to the WNV isotype control mAb at 2 dpi or 28 dpi. Data represents two (C-F), three (B) or four (A) independent experiments. The dotted line represents the limit of detection. Data in A and B were analyzed using an unpaired, two-tailed Student’s *t* test. All error bars indicate SEM. Data in E and F were analyzed using an unpaired, two-tailed Student’s *t* test (*, *P* < 0.05).

Chronic CHIKV RNA can consistently be detected by RT-qPCR in animal models, including the joints and spleen of mice and the spleen, lymph nodes, and liver of macaques [34,41]. However, techniques to visualize this persistent RNA have been inadequate. Previous reports have suggested that viral RNA and proteins may reside within synovial macrophages or endothelial cells [18,34,35] although the cell types harboring persistent CHIKV RNA have not been rigorously examined. To determine if the tdTomato^+^ fibroblasts in the ankle and skin are enriched for persistent CHIKV RNA, we sorted the CD45^-^ cells isolated from the ankle and skin at 28 dpi into tdTomato^+^ and tdTomato^-^ populations, extracted the RNA from these cells, and then performed RT-qPCR for CHIKV E1 RNA. In both the skin and ankle tissue CHIKV RNA was enriched in the tdTomato^+^ population compared to the tdTomato^-^ population (**Fig 8A and 8B**). In the skin, there was approximately an 8-fold enrichment for E1 RNA levels in the tdTomato^+^ cells compared to the tdTomato^-^ cells, where the RNA levels cells were near the limit of detection (**Fig 8A**). Similarly, the levels of E1 RNA in the ankle were 4-fold higher in the tdTomato^+^ population compared to the tdTomato^-^ cells (**Fig 8B**). Overall, this demonstrates that tdTomato^+^ cells in the skin and ankle that survive acute CHIKV infection harbor persistent CHIKV RNA during chronic disease.

Since the tdTomato^+^ cells harbor chronic viral RNA we next evaluated these cells for surface expression of the CHIKV E1 and E2 proteins. Reporter mice were infected with CHIKV-3’-Cre and the ankle tissue was harvested at either acute (2 dpi) or chronic (28 dpi) time points. The isolated cells were stained for CD45, CD29, CD44 and with anti-CHIKV E1 and E2 mAbs [60] or with an anti-WNV mAb [61] as an isotype control. At 2 dpi, in the ankle we detected between 2–6% of the CD29^+^CD44^int^ fibroblasts (approx. 1.1x10^5^ cells) that stained positively with the anti-CHIKV E1 and E2 antibody (**Fig 8C and 8E**). Of the cells isolated from the skin, less than 0.5% of cells were positive for CHIKV E1 and E2 at 2 dpi, so we focused our analysis on the ankle. At 2 dpi, tdTomato expression was not yet detected (**Fig 8C and 8F**). An analysis of CD44^int^ CD29^+^ fibroblasts at 28 dpi revealed no cell surface expression of CHIKV E1 and E2 (**Fig 8D and 8E**). If we gated on all of the tdTomato^+^ cells isolated from the ankle at 28 dpi, which accounted for less than 1% of the cells isolated, we identified less than 1% expressing CHIKV E1 and E2 on the cell surface, accounting for less than 0.01% of all cells isolated from the ankle (**Fig 8D and 8F**). Therefore, while these tdTomato^+^ cells survive acute CHIKV infection and harbor persistent viral RNA, the CHIKV E1 and E2 proteins are not expressed on the cell surface of the majority of cells.

## Discussion

CHIKV causes a debilitating acute disease that results in chronic arthralgia and myalgia in a substantial proportion of patients. The mechanism of this chronic CHIKV pathogenesis is unclear but may be related to persistence of viral RNA in musculoskeletal tissues. To begin to identify the cells that contribute to chronic pathogenesis, we created a recombinant CHIKV that expresses Cre recombinase and permanently marks infected cells in reporter mice. Using this tool, we provide evidence that a subset of cells survive CHIKV infection, are present during the chronic stages of disease, and harbor persistent CHIKV RNA. Previous reports have used immunofluorescence microscopy, immunohistochemistry, or RNA quantification to detect CHIKV antigen or RNA in animal and patient cells during the chronic phase of disease [18,33–35]. However, CHIKV antigen-positive cells are reportedly rare in samples from the subacute or convalescent phase, likely owing to the insensitivity of these techniques. We used our system to elucidate the cells that survive acute infection and determine whether they are reservoirs of chronic CHIKV RNA.

We constructed two variants of the CHIKV-Cre construct, inserting the Cre recombinase gene under control of a second subgenomic promoter, either in between the structural and non-structural genes (CHIKV-5ʹ-Cre) or at the 3ʹ end of the genome (CHIKV-3ʹ-Cre). As has been reported previously with CHIKV-GFP clones [62,63], the introduction of Cre recombinase mildly attenuated both viruses. However, the CHIKV-Cre viruses retained sufficient virulence to infect targets of CHIKV replication (fibroblasts and myoblasts) and induce acute swelling and histopathology that was comparable to that induced by CHIKV-WT. The CHIKV-Cre viruses also established chronic disease with persistence of viral RNA, chronic myositis, and synovial inflammation. Using this CHIKV-Cre and tdTomato reporter mice, we showed that, like viral RNA, tdTomato^+^ cells can be detected in the foot and ankles of infected mice for at least 112 days (16 weeks) after infection.

One limitation of the model is that we cannot be certain at what stage a given tdTomato^+^ cell became infected with CHIKV. Based upon the presence of infectious viral titers and the increased E1:nsP1 ratio that is detected only during acute infection, we favor a model in which cells are infected during the first week of infection, survive acute infection, and persist for the lifetime of the cell. Our current model cannot exclude other possibilities including that some tdTomato^+^ cells arise as daughter cells from mitotic replication of directly infected tdTomato^+^ cells or that tdTomato^+^ cells arise throughout acute and chronic phases via ongoing infection with low levels of replicating virus, that to date has been undetectable.

For our more detailed studies, we used the CHIKV-3ʹ-Cre rather than CHIKV-5ʹ-Cre, as certain aspects of CHIKV-3ʹ-Cre infection more closely resembled CHIKV-WT infection. For example, the ratio of E1:nsP1 in the first seven days of infection was more similar between CHIKV-3ʹ-Cre and CHIKV-WT than with CHIKV-5ʹ-Cre. This difference is likely because the E1 gene is under the replicative direction of the subgenomic promoters; the internal genomic structure of CHIKV-3ʹ-Cre is identical to CHIKV-WT in contrast to CHIKV-5ʹ-Cre.

Using our CHIKV-3ʹ-Cre and tdTomato reporter mouse system, we identified cell types targeted by CHIKV that persist into the chronic phase. In the acute phase, CHIKV has been shown to infect fibroblasts, synoviocytes, macrophages, skeletal muscle fibers, satellite cells, osteoblasts, endothelial cells, keratinocytes, and neurons [19,33,47,48]. *In vitro* infections with many of these cell types exhibit high levels of cytopathic effects and cell death [57,58]. Immunofluorescence analysis using our reporter mice showed that the persistent tdTomato^+^ cells are predominantly a mixture of SAA^+^ skeletal myofibers and vimentin^+^ CD44^+^ CD29^+^ cells that are likely fibroblasts; both of these cell types have been reported as permissive for CHIKV infection during the acute phase [46,47,49]. Identification of tdTomato^+^ CD44^int^ CD29^+^ fibroblasts was confirmed by flow cytometry in both the skin and ankle tissue. In the skin, using markers for fibroblasts, endothelial cells, and immune cells we could identify approximately 90% of the tdTomato^+^ cells, with CD44^int^ CD29^+^ fibroblasts accounting for 70–80% of all tdTomato^+^ cells isolated. In the ankle tissue, we only identified approximately 75% of the uninuclear cells marked during chronic CHIKV, with the majority once again being fibroblasts. Therefore, 25% of the tdTomato^+^ cells are unidentified in the ankle. Our digests and preparation did not allow us to isolate the muscle fibers so they are not included in our analysis. Additional cell types such as muscle satellite cells are known to be infected by CHIKV and could contribute to the tdTomato^+^ population [33]. Further studies will be needed to identify these additional cell types.

Other cell types such as endothelial cells and macrophages also have been reported to contain CHIKV antigens during chronic infection in humans, macaques, and mice [18,34,35]. Some reports also have suggested that hematopoietic cells such as monocytes or macrophages can be directly infected by CHIKV [47,64]. In our immunofluorescence analysis, the vast majority of tdTomato^+^ cells were CD31^-^ and CD45^-^, which is consistent with previous reports examining CHIKV staining in acute skin samples [46]. However, flow cytometric analysis revealed a small population, less than 15% of all tdTomato^+^ cells, which were CD45^+^ in the ankle and skin tissues. We also found that less than 5% of the tdTomato^+^ cells were CD31^+^. The CD45^+^ tdTomato^+^ cells expressed a lower tdTomato MFI than the CD45^-^ tdTomato^+^ population. The reason for the difference in MFI is unclear. There are mixed reports on whether macrophages can be productively infected by CHIKV [47,64]. It is possible that rather than being directly infected, these phagocytic cells have internalized viral RNA from adjacent tdTomato^+^ cells. Further studies will be needed to evaluate this possibility.

Not all cells known to be infected by CHIKV in the acute phase were apparent as tdTomato^+^ in our analysis at chronic time points. Few cells in the synovial membrane were tdTomato^+^, even though synovial inflammation was present during acute disease and mild inflammation persisted at chronic time points, as has been reported [39,41]. During the acute stage of infection, we could detect staining for viral RNA in the synovium; however, the synovial staining was less pronounced than the staining seen in the muscle or skin for both CHIKV-WT and CHIKV-3ʹ-Cre. These results may indicate that synovial cells are not infected in large numbers *in vivo*, or that synovial cells are infected but do not survive. These findings could explain why previous efforts to detect viral components in patient samples at chronic phase time points generally have been unsuccessful, as they have focused on synovial fluid analysis and occasionally synovial tissue but rarely skin or muscle biopsies. In addition to a paucity of tdTomato^+^ synovial cells, few tdTomato^+^ cells were present in or associated with bone. A small number of tdTomato^+^ cells occasionally were found associated adjacent to the periosteum, which could represent a population of fibroblasts or osteoblasts. Previous reports have suggested that CHIKV infection of osteoblasts can perturb osteoclast function and lead to chronic histological damage [65,66]. These findings are not precluded by our results, as osteoblasts may accomplish such outcomes while still succumbing to lytic infection.

A key unanswered question in CHIKV disease pathogenesis is what mechanistically promotes symptoms during the chronic phase. Replicating virus has not been detected at chronic time points, yet chronic inflammation is clearly present in tissue, serum, and synovial samples, both histologically and transcriptionally [67–69]. Although viral RNA can be detected in the chronic phase, to date it has been difficult to identify the cells harboring it. The treatment of mice with anti-Mxra8 mAb not only reduced the total number of tdTomato^+^ cells by 75%, but also decreased the levels of chronic RNA by 73%, thus correlating the level of chronic viral RNA and the number of persistent tdTomato^+^ cells in the chronic phase. Such results suggested that persistent viral RNA might be harbored within the tdTomato^+^ cells. Our model allowed us to isolate uninuclear CD45^-^ tdTomato^+^ cells in the ankle and skin tissue and definitively show that they harbor persistent CHIKV RNA at chronic phase time points. Despite evidence that the skin and muscle fibroblasts harbor viral RNA at these time points, we were unable to detect the expression of viral CHIKV surface proteins in the vast majority of cells examined. Gating on the tdTomato^+^ cells did allow us to identify a rare population of cells (fewer than 1% of all tdTomato^+^ cells) that expressed cell surface CHIKV antigens. At this time it is unclear if the chronic viral RNA we detected is located in only these rare CHIKV^+^ cells or if the majority of cells harboring viral RNA do not express CHIKV antigens. Single cell RNA sequencing analysis will be needed to further clarify these findings. In addition, since muscle fibers could not be isolated and analyzed by flow cytometry, it is unknown if they harbor persistent CHIKV RNA and/or express cell surface CHIKV antigens. How this viral RNA is able to persist in these cells, why the majority of these cell do not express CHIKV antigens on their cell surface, and whether these tdTomato^+^ cells are transcriptionally altered and ultimately drive chronic pathogenesis of CHIKV are areas of future investigation.

In conclusion, our CHIKV-3ʹ-Cre and tdTomato system provides further evidence for musculoskeletal cells as targets of CHIKV infection in the acute and chronic stages of disease and revealed that fibroblasts that survive acute CHIKV harbor persistent CHIKV RNA. How these cells contribute to pathogenesis remains to be elucidated. Uncovering the mechanisms for long-term pathogenesis could aid in the development of treatments and preventative measures for this incapacitating, virally-induced chronic arthritis.

## Materials and methods

### Viruses

The wild-type strain of CHIKV (denoted CHIKV-WT) used in these studies is LR2006 OPY1, an ECSA strain of CHIKV isolated from the La Réunion Island outbreak [62]. Plasmids for CHIKV-5ʹ-GFP and CHIKV-3ʹ-GFP were obtained from Stephen Higgs (Kansas State University), and the Cre recombinase gene, with a nuclear localization signal (NLS) sequence, was substituted for GFP in both viruses [62,63]. To produce the CHIKV-5ʹ-Cre plasmid, Cre was PCR amplified from a Cre-containing plasmid with a forward primer (FW) containing an AscI restriction site and homology to the 5ʹ end of the Cre gene and a reverse primer (RV) containing a PmeI site and homology to the 3ʹ end of the Cre gene. To produce the CHIKV-3ʹ-Cre plasmid, Cre was PCR amplified from the CHIKV-5ʹ-Cre plasmid using AscI-Cre FW and BsmBI-Cre RV primers. The 3ʹ-UTR also was PCR amplified from the CHIKV-3ʹ-GFP virus using BsmBI-3ʹUTR FW and NotI-3ʹ-UTR RV primers. Both PCR fragments were inserted concurrently into a CHIKV-3ʹ-GFP plasmid linearized with AscI and NotI. The infectious clone plasmids for CHIKV-WT, CHIKV-3ʹ-Cre, and CHIKV-5ʹ-Cre were sequenced and had 100% nucleotide identity with reference and predicted sequences.

To produce recombinant viral stocks, the infectious clone plasmids were linearized with NotI, and RNA was produced using a SP6 DNA-dependent RNA transcription kit (Agilent, Promega, BioLabs). The RNA was transfected into baby hamster kidney cells (BHK-21 cells) using Lipofectamine 2000 (Invitrogen), and after 48 h the supernatant was collected, centrifuged at 150–300 x g to clear cell debris, aliquoted, and stored at -80°C. Titers of the viral stocks were assessed by plaque assay as previously described [70]. All cell and animal work with live CHIKV was performed in a biosafety level 3 facility and followed guidelines established by the Environmental Health and Safety Committee at Washington University School of Medicine.

### Mice

The following strains of mice were obtained from the Jackson Laboratory: C57BL/6 (JAX Stock No: 000664; C57BL/6J) and tdTomato reporter mice (JAX Stock No: 007914; B6.Cg-*Gt(ROSA)*^*26Sortm14(CAG-tdTomato)Hze*^/J). Mice were bred and maintained at Washington University School of Medicine animal facilities in accordance with all federal and University guidelines. For all experiments, mice were weight- and sex-matched prior to infection; both sexes were used. For mouse studies, the principles of Good Laboratory Animal Care were adhered to in strict accordance with NIH recommendations [71]. All animal protocols were approved by the Animal Studies Committee at Washington University. Every effort was made to minimize animal suffering.

### Cells and media

Murine embryonic fibroblasts (MEFs) were generated from C57BL/6 and tdTomato reporter mice and grown at 37°C and 5% CO_2_ in CD10 media: Dulbecco’s modified Eagle medium (DMEM) (Corning) supplemented with 10% fetal bovine serum (FBS) (Biowest), 1% penicillin-streptomycin (P/S; Corning), 1% L-glutamine (Glu; Corning), 1% non-essential amino acids (NEAA; Corning), and 1% HEPES (Corning). For some experiments CD1 media (CD10 media with only 1% FBS) was used. MEFs were used prior to passage number 5 for these studies. The C2C12 myoblast cell line was obtained from ATCC (ATCC CRL1772) and cultured using the same conditions as the MEFs.

### Viral growth curves

For viral growth curves, MEFs were plated at 2 x 10^4^ cells/well and C2C12s at 1 x 10^4^ cells/well in 96-well tissue culture-treated plates and allowed to adhere overnight. Cells were rinsed and diluted virus was then added at the indicated MOI and allowed to adhere for 1 h. The input virus was then removed, the cells were washed once, and fresh CD1 media was added. At each time point, a plate was frozen at -80°C and underwent three freeze-thaw cycles before titers were determined on BHK-21 cells by plaque assay.

### *In vitro* coverslip studies

tdTomato reporter MEFs were plated onto collagen type I-treated 22 mm glass coverslips (Corning) at 4 x 10^5^ cells/well in 6-well tissue culture-treated plates in CD10. If indicated, cells also were concurrently pre-treated with ~100 U IFN-β (PBL Assay Science), and all cells were allowed to adhere to the coverslips overnight. Cells were inoculated with virus at the indicated MOI, as described above. After 48 h, the supernatant was removed, and the cells were fixed to the coverslips with 4% paraformaldehyde (PFA) for at least 10 min. Coverslips were stored in the wells with phosphate-buffered solution (PBS) at 4°C until further processing.

Coverslips were prepared for microscopy as follows: coverslips were permeabilized for 10 min at room temperature (RT, ~23°C) with 0.2% Triton-X in PBST (PBS + 0.1% Tween20), washed with PBST, stained with DAPI (100 **μ**g/mL in PBST) for 10 min at RT, washed with PBST, mounted onto a glass slide using ProLong Gold (Invitrogen), and allowed to cure protected from light at RT overnight. Coverslips were imaged using a Nikon Spinning Disk Confocal Microscope, maintained at the Washington University Center for Cellular Imaging. For illustration purposes, cells were imaged at 4x or 10x with a single capture using the DAPI and RFP channels. For quantification purposes, four random locations were selected on each coverslip, and a 4x4 stitched image of 16 adjacent 4x images (15% overlap) was produced from each location. Image files were processed and automatically quantified for cell number and tdTomato^+^ cell count using ImageJ. The nuclei or cells were quantified using the Quantify Particles function, with the size being a minimum of 500 pixels^2^. The DAPI^+^ nuclei count represents the total number of cells per field, and the RFP^+^ cell count represents the total number of tdTomato^+^ cells per field. The percentage of tdTomato^+^ cells was calculated by dividing the total number of tdTomato^+^ cells per field by the total number of nuclei per field for each image.

### Viral burden studies in animals

Four-week old C57BL/6 mice sedated with ketamine were injected subcutaneously with 10^5^−10^6^ plaque-forming units (PFU) of virus diluted in 10–30 **μ**L of CD1 without P/S into the left (ipsilateral) footpad, between the second and third digits. Swelling of the infected foot was monitored daily using digital calipers by measuring both height and width. For infectious virus and viral RNA samples, mice were sedated and sacrificed with ketamine at the indicated time point and were perfused with 5–10 mL PBS. Tissues (spleen, ipsilateral and contralateral ankles with toes removed and skin included, and ipsilateral quadriceps muscles) were harvested into homogenization bead tubes containing 600 **μ**L of PBS and stored at -80°C. Infectious virus titer was determined by plaque assay. For viral RNA samples, bead tubes containing organ samples were snap frozen in liquid nitrogen before transfer to -80°C. Viral RNA levels were determined using RT-qPCR, as described below. Blood samples were allowed to coagulate at RT, samples were centrifuged at 9,000 x g for 5–10 min, and serum was transferred to a fresh tube and stored at -80°C.

For Mxra8 mAb studies, tdTomato reporter mice were inoculated with CHIKV-3ʹ-Cre as described above. The mice were injected via an intraperitoneal route with 250 **μ**g of Armenian hamster isotype control (Bio X Cell # BE0260) or Armenian hamster anti-Mxra-8 mAbs diluted in PBS (125 **μ**g each of 1G11.E6 + 7F1.D8) [59] at 12 h prior to infection and at 4, 8, 12, 16, 20, and 24 dpi. Samples were harvested for RNA or histology as described above. RT-qPCR analysis, frozen section slides and quantification, and immunofluorescence was performed as described below.

### Quantitative real-time PCR

RNA was isolated from Trizol-tissue homogenates using the standard Trizol-chloroform extraction protocol and/or an RNAasy mini-prep kit (Qiagen). For analysis of sorted cells, RNA was isolated using RNeasy FFPE Kite (Qiagen). Modifications to the suggested protocol include elimination of all steps for processing FFPE tissues such as deparaffinization. Isolated RNA was resuspended in UltraPure H_2_O (Ambion) and stored at 4°C overnight or -80°C for long-term storage. Viral standards were generated by producing RNA from the infectious clone plasmids as described above. The copy number of the standard was determined by quantifying the sample and calculating the copy number using the known genome length. A 1:10 dilution series of the standard was prepared ranging from ~10^1^ copies to ~10^9^ copies. RT-qPCR was performed on a Bio-Rad CFX machine using the TaqMan RNA-to-CT 1-Step Kit (Applied Biosystems) with a 25 **μ**L total reaction volume per well, 2 **μ**L of RNA sample, and the indicated amount of Taqman primers and probe from Integrated DNA Technologies: E1 FW (5ʹ- TCG ACG CGC CCT CTT TAA -3ʹ), E1 probe (5ʹ- /56-FAM/ ACC AGC CTG/ ZEN/ CAC CCA TTC CTC AGA C/ 3IABkFQ/ -3ʹ), E1 RV (5ʹ- ATC GAA TGC ACC GCA CAC T -3ʹ), nsP1 FW (5ʹ- AAA GGG CAA ACT CAG CTT CAC -3ʹ), nsP1 probe (5ʹ-/ 56-FAM/ CGC TGT GAT/ ZEN/ ACA GTG GTT TCG TGT G/ 3IABkFQ/ -3ʹ), nsP1 RV (5ʹ- GCC TGG GCT CAT CGT TAT TC -3ʹ). For RNA isolated from sorted cells, 9 **μ**L of RNA was analyzed by RT-qPCR in a 25 **μ**L total reaction volume. To quantify the Cre copy number, pre-made Enterobacteria phage P1 cyclization recombinase FAM Taqman copy number primers/probe were used (ThermoFischer Mr00635245_cn), and 1.25 **μ**L of the 20x working stock was used per 25 **μ**L reaction. The following PCR cycling protocol was used: 30 minutes at 48°, 10 min at 95°, and 40 cycles of 15 sec at 95° and 1 min at 60°C. RNA copy number was determined for each sample using the matching RNA standard, and the copy number was normalized to the total **μ**g of RNA for each sample (determined by NanoDrop).

### Histology studies

Four-week old tdTomato mice were infected as described above. For histology samples, mice were sedated with ketamine and sacrificed at the indicated time point, and 5–10 mL of 4% PFA was perfused into the heart. Tissues (*e*.*g*., spleen, ipsilateral and contralateral feet/ankles) were harvested and fixed in 4% PFA. After 48 h of immersion fixation, tissues were washed with PBS and transferred to BSL2 facilities for further processing. Tissues containing bone (*e*.*g*., whole foot/ankle samples) were decalcified in 14% acid-free EDTA (VWR) for 14 days. For frozen section processing, decalcified tissues and soft tissues (*e*.*g*., spleen) were equilibrated overnight in 30% sucrose in PBS and then frozen in optimal cutting temperature (OCT) compound (Tissue Tek). Samples were then cut with a cryostat at 10 **μ**m for spleens or 30 **μ**m for foot/ankle samples onto SuperFrost Plus slides (Fisher). Slides were stored at -20°C until further processing.

Frozen section slides were prepared for microscopy by fixing with cold acetone, permeabilizing with 0.2% Triton-X in PBST, washing with PBST, and mounting with a No. 1-1/2 glass coverslip (VWR) using Vectashield containing DAPI (Vector Laboratories H-1200). Slides were imaged using a Nikon Spinning Disk Confocal Microscope, maintained at the Washington University Center for Cellular Imaging. Whole tiled images of the foot and ankle were prepared using the 4x objective and the DAPI, GFP, and RFP channels using large-image capture and 15% overlap. Alternatively, frozen section slides were processed and imaged using immunofluorescence, as described below. Image files were processed using ImageJ. The total number or tissue-specific number of tdTomato^+^ cells was quantified by eye using manual cell counters in ImageJ or Photoshop; quantification was performed in a blinded manner. The total area of each foot/ankle or tissue was measured using ImageJ, and each count of total tdTomato^+^ cells was normalized to the respective area.

For paraffin section processing, decalcified tissues were dehydrated using washes of 30% ethanol (EtOH), 50% EtOH, and 70% EtOH. Samples were submitted to the Musculoskeletal Research Center for paraffin embedding, sectioning, and hematoxylin and eosin (H&E) staining. Histopathologic examination and analysis was performed by a pathologist blinded to intervention group or time point. The presence of acute and/or chronic inflammation within the skeletal muscle, synovial tissues, and joint space was noted. The overall severity of inflammation was scored as follows: 0 for none, 1 for mild, 2 for moderate, and 3 for severe. Representative images were taken using the Zeiss Axio Imager Z2 Fluorescence Microscope with ApoTome 2, managed at the Washington University Center for Cellular Imaging.

### Immunofluorescence

Frozen section slides were first fixed with cold acetone. If indicated for the specific antibody, the samples underwent antigen retrieval using antigen unmasking solution (H3300; Vector Laboratories), which was incubated overnight in a 60°C water bath, protected from light. Samples were then permeabilized with 0.2% Triton-X in PBST, washed with PBST and then blocked for 1–2 h with TSA blocking reagent (Perkin Elmer FP1012). Samples were then incubated overnight at 4°C in primary antibody diluted 1:100 in 3% normal goat serum (NGS, Equitech-Bio, Inc.) and 3% bovine serum albumin (BSA, Sigma) in PBST. After two washes with PBST, samples were incubated for 1–2 h at RT in secondary antibody diluted 1:500 in 1% BSA PBST. Samples were washed once with PBST, stained with DAPI (100 **μ**g/mL in PBST) for 10 min at RT, washed once with PBST, and a glass coverslip was mounted and slides were allowed to cure protected from light at RT overnight. Slides were imaged using a Nikon Spinning Disk Confocal Microscope maintained at the Washington University Center for Cellular Imaging. For illustration purposes, cells were imaged at 20x or 100x with a single capture using the DAPI, GFP, RFP, and or AF647 channels.

The following primary antibodies were used for these studies with antigen retrieval: anti-vimentin (Abcam, clone EPR3776); anti-CD45 (Abcam, polyclonal ab10558); anti-CD44 (Abcam, clone EPR18668); anti-CD29 (eBioscience, clone KMI6). The following antibodies did not require antigen retrieval: anti-CD31 (BD Biosciences, clone MEC 13.3); anti-SAA (Abcam, clone EA-53).

### Flow cytometry and florescence activated cell sorting

Mice were sacrificed 28 dpi and perfused with PBS. To isolate cells from the skin, the toes were removed, and the cutaneous and subcutaneous tissues were everted and minced. In addition, the ankle tissue, without the skin, was disarticulated without fracturing the bone. Tissues were individually incubated for 2 h at 37°C in 5 mL digestion buffer with manual shaking every 20–30 min. Digestion buffer consisted of RPMI (Sigma), type IV collagenase (2.5 mg/mL, Sigma), Liberase TL (100 **μ**g/mL, Roche), DNase I (10 mg/mL, Sigma), 15 mM HEPES (Corning), and 10% FBS (BioWest). Digested tissues were passed through a 70 **μ**m cell strainer and washed once with 40 mL PBS containing 5% FBS (FACS buffer). The number of viable cells was quantified by trypan blue staining.

The single cell suspension was transferred to a 96-well plate and incubated with anti-mouse CD16/CD32 (Clone 93; BioLegend) for 10 min at 4°C and then surface-stained in PBS containing 5% FBS for 1 h at 4°C. All antibodies were diluted 1:200 and are from BioLegend unless otherwise specified: anti-CD45 FITC (30-F11), Fixable Viability Dye eFluor 506 (1:500, eBioscience), anti-CD29 PerCP-Cy5.5, anti-CD44 PE-Cy7, and anti-CD31 APC. Viral CHIKV antigens E1 and E2 were detected on the cell surface using N297Q biotinylated humanized CHK-152 (5 **μ**g/mL) and murine CHK-166 (5 **μ**g/mL) mAbs [60], with a N297Q biotinylated humanized West Nile virus (WNV) E16 serving as the isotype control. Secondary staining was followed with streptavidin-conjugated APC (1:500) (BioLegend). Gating strategy for ankle and skin cells is shown in **S6A and S6D Fig**.

After staining, cells were washed and fixed at 4°C for 10 min in 4% paraformaldehyde (Electron Microscopy Sciences). The fixed cells were washed and resuspended in PBS containing 5% FBS. Cells were processed on a LSR Fortessa (Becton Dickinson) flow cytometer or sorted using a FACSAria II (Becton Dickinson) managed by the Flow Cytometry & Fluorescence Activated Cell Sorting Core at Washington University and analyzed using BD FACSDiva and FlowJo v10 software (Tree Star Inc.).

### RNA *in situ* hybridization

Paraffin sections were processed using the provided RNAscope reagents and protocols from Advanced Cell Diagnostics. Slides were first prepared as directed for formalin-fixed paraffin-embedded (FFPE) samples (ACD Document Number 322452). The prepared samples were then exposed to RNAscope Probe V-CHIKV-sp (ACD 479501). The probed samples were then detected following the RNAscope 2.5 HD Detection Reagent–BROWN protocol (ACD Document Number 322310), with the only modification being that slides were washed via pipetting the wash buffer onto the slides instead of submerging the slides into wash buffer. Representative images were taken using the Zeiss Axio Imager Z2 Fluorescence Microscope with ApoTome 2, managed at the Washington University Center for Cellular Imaging.

### Statistical analysis

All data were analyzed using the Prism software, version 7 (GraphPad), as detailed in the figure legends. The following statistical tests were used: two tailed Student’s unpaired *t* test, Student’s paired *t* test, Mann-Whitney test, ordinary one-way analysis of variance (ANOVA), ordinary two-way ANOVA, and two-way repeated-measures ANOVA. The following post-tests for multiple comparisons were also used: Bonferroni’s post-test, Dunnett’s post-test, Tukey’s post-test, and Sidak’s post-test. All error bars indicate standard error of the mean (SEM); if error bars are not visible, then they are shorter than the height of the symbol. Asterisks indicate statistical significance, with only relevant comparisons shown (*, *P* < 0.05; **, *P* < 0.01; ***, *P* < 0.001; ****, *P* < 0.0001).

### Ethics statement

Experiments were approved and performed in accordance with the recommendations in the Guide for the Care and Use of Laboratory Animals of the National Institutes of Health. The protocols were approved by the Institutional Animal Care and Use Committee at the Washington University School of Medicine (Assurance number A3381-01).

## Supporting information

S1 Fig CHIKV-5ʹ-Cre and CHIKV-3ʹ-Cre mark reporter cells *in vitro* and grow productively in muscle cells and fibroblasts. **(A)** Genome maps of the three CHIKV clones that were used in these studies: CHIKV-WT, CHIKV-3ʹ-Cre, and CHIKV-5ʹ-Cre. **(B)** tdTomato MEFs plated in 96-well plates (2.5 x 10^4^ cells/well) were inoculated with CHIKV-5ʹ-Cre (purple inverted triangles) or CHIKV-3ʹ-Cre (red triangles) at an MOI of 0.1, 1.0, or 3.0 and analyzed for fluorescence. **(C)** tdTomato MEFs plated in 96-well plates (2.5 x 10^4^ cells/well) and were mock-infected (mock) or infected at an MOI of 3.0 with CHIKV-WT (WT), CHIKV-5ʹ-Cre (5ʹ Cre), or CHIKV-5ʹ-Cre pretreated with ~100 U IFN-β (5ʹ Cre + IFN-β). **(D)** Representative growth curves of C2C12 myoblasts or **(E)** C57BL/6 MEFs infected with CHIKV-WT (green circles), CHIKV-5ʹ-Cre (purple inverted triangles), or CHIKV-3ʹ-Cre (red triangles) at an MOI of 0.05. The number of tdTomato^+^ cells per well in **B** and **C** were quantified at 2 dpi manually using a fluorescent microscope and are representative of two independent experiments. Data in **B** were analyzed with a two-way ANOVA using Sidak's post-test. Data in **C** were analyzed with an ordinary one-way ANOVA using Sidak’s post-test. All error bars indicate SEM. (*, *P* < 0.05; **, *P* < 0.01; ***, *P* < 0.001; ****, *P* < 0.0001).
(TIF)

S2 Fig Characterization of clinical disease and viral replication of CHIKV-5ʹ-Cre and CHIKV-3ʹ-Cre viruses. **(A)** Swelling of ipsilateral feet of mice inoculated with 10^6^ PFU of CHIKV-WT (open green circles; also shown in Fig 2A) or 10^6^ PFU of CHIKV-5ʹ-Cre (purple). Data were pooled from two independent experiments with n = 10 for each virus. **(B-F)** Levels of infectious virus in mice infected with 10^6^ PFU of CHIKV-WT (solid green circles; or open green circles in S2B, also shown in Fig 2B), 10^6^ PFU of CHIKV-5ʹ-Cre (purple inverted triangles), or 10^6^ PFU of CHIKV-3ʹ-Cre (red triangles) in **(B)** ipsilateral ankle, **(C)** serum, **(D)** ipsilateral quadriceps muscle, **(E)** the contralateral ankle, or **(F)** spleen. For **B-F**, each time point for each virus and organ represents 5–7 mice and were pooled from at least 2 independent experiments. Infectious virus levels during acute infection was measured by plaque assay, normalized to gram of tissue, and then log-transformed. The dashed line for **B-F** represents limit of detection for the plaque assay. Data in B-F were log-transformed prior to analysis. Data in A were analyzed with a two-way repeated measures (RM) ANOVA with Bonferroni’s post-test, and data in **B-F** were analyzed with an ordinary two-way ANOVA. Sidak's post-test was used for **A**, and **B**; Dunnett's post-test comparing WT as the control column was used for **C-F**. All error bars indicate SEM. (*, *P* < 0.05; **, *P* < 0.01; ***, *P* < 0.001; ****, *P* < 0.0001).
(TIF)

S3 Fig CHIKV-5ʹ-Cre and CHIKV-3ʹ-Cre retain their pathogenic properties to induce acute arthritis. **(A)** Mice were mock-infected or infected with 10^6^ PFU CHIKV-WT (WT) or CHIKV-3ʹ-Cre (3ʹ-Cre), and ipsilateral ankles were probed for CHIKV RNA using *in situ* hybridization at 2 dpi. Paraffin sections were stained with a probe for E1 CHIKV-LR RNA as outlined in the Methods. Representative images are shown of the skin, muscle, and synovium. Scale bars represent 100 μm. Data represents two independent experiments with 6 mice per virus and 2 mock-infected mice. **(B-C)** Mice were mock-infected (mock, blue diamonds) or inoculated with 10^6^ PFU CHIKV-WT (WT, green circles) or CHIKV-5ʹ-Cre (5ʹ-Cre, purple inverted triangles), and ipsilateral ankles were taken for H&E histology at 7 dpi. **(B)** Representative images are shown of the skin, muscle, and synovium from CHIKV-5ʹ-Cre infected samples; scale bar represents 100 μm. The skin and associated tissue is divided (from left to right) into muscle (M), hypodermis (H), dermis (D), and epidermis (E). The muscle section is divided into tendon (T) and muscle (M). The synovium section shows synovium (S) and bone (B), with asterisks indicating synovial inflammation and arrows indicating immune infiltrates into the synovial cavity. **(C)** Ankles from **B** were scored for overall histological damage, compared to mock-infected and CHIKV-WT-infected samples. Open symbols for mock and WT indicate that these data are also shown in the corresponding Fig 2C graph. Samples were pooled from two independent experiments. Data in **C** were statistically analyzed with a one-way ANOVA with Tukey's post-test. All error bars indicate SEM. (*, *P* < 0.05; **, *P* < 0.01; ***, *P* < 0.001; ****, *P* < 0.0001).
(TIF)

S4 Fig CHIKV-5ʹ-Cre and CHIKV-3ʹ-Cre retain their pathogenic properties to induce chronic disease. **(A-B)** Mice were mock-infected (mock, blue diamonds) or infected with 10^6^ PFU CHIKV-WT (WT, green circles) or CHIKV-5ʹ-Cre (5ʹ-Cre, purple inverted triangles), and at 28 dpi ipsilateral ankles were analyzed histologically after hematoxylin and eosin staining of sections. **(A)** Representative images are shown of the skin, muscle, and synovium from CHIKV-5ʹ-Cre samples; scale bar represents 100 μm. The skin and associated tissue is divided (from left to right) into muscle (M), hypodermis (H), dermis (D), and epidermis (E). The muscle section is divided into muscle (M) and tendon (T), with black ovals around focal patches of cellular filtrates. The synovium section shows synovium (S) and bone (B), with asterisks indicating synovial inflammation and proliferation. **(B)** Ankles from A were scored for overall histological damage, compared to mock-infected and CHIKV-WT-infected samples. Open symbols for mock and WT indicate that these data are also shown in Fig 3B graph. **(C)** RNA was isolated from the ipsilateral ankles of mice inoculated with 10^6^ PFU of CHIKV-WT (open green circles, also shown in Fig 3D) or 10^6^ PFU of CHIKV-5ʹ-Cre (purple inverted triangles) at time points ranging from 0 to 112 dpi and analyzed by RT-qPCR for viral E1, nsp1, or Cre RNA copy number. Samples were normalized to total μg of RNA isolated for each sample and then log-transformed. **(C)** Viral E1 RNA levels were measured. **(D)** The ratio of viral E1 RNA to viral nsP1 RNA in the same samples as C (open green circles, also shown in Fig 3E). **(E)** The ratio of viral Cre RNA to viral nsP1 RNA in mice inoculated with 10^6^ PFU of CHIKV-5ʹ-Cre (purple inverted triangles) or CHIKV-3ʹ-Cre (red triangles). Data in **B** were analyzed with a one-way ANOVA with Tukey's post-test. For **C-E**, each time point for each virus represents 4–20 mice and was pooled from at least 2 independent experiments. The dashed line in **C** represents the limit of detection for the RT-qPCR assay; for **D** and **E**, the dotted line represents a ratio of 1. Data in **C-E** were analyzed with a two-way ANOVA with Sidak’s post-test. All error bars indicate SEM. (*, *P* < 0.05; **, *P* < 0.01; ***, *P* < 0.001; ****, *P* < 0.0001).
(TIF)

S5 Fig Cells are marked by CHIKV-5ʹ-Cre or CHIKV-3ʹ-Cre at chronic time points. **(A)** C57BL/6 or **(B)** tdTomato mice were inoculated with 10^6^ PFU CHIKV-WT (WT, green circles), CHIKV-5ʹ-Cre (5ʹ-Cre, purple inverted triangles), or CHIKV-3ʹ-Cre (3ʹ-Cre, red triangles). Ipsilateral ankles were processed at 28 dpi, and the total number of tdTomato^+^ cells in frozen tissue sections was quantified. Open green circles for WT and open red triangles for 3ʹ-Cre indicate that these data are also shown in the corresponding Fig 4B graph. **(C)** Representative image of a whole foot/ankle from a tdTomato mouse infected with 5ʹ-Cre at 28 dpi. Blue shows DAPI staining, and red is tdTomato; scale bars represent 1000 μm. **(D)** Time course of tdTomato mice infected with CHIKV-5ʹ-Cre (purpled inverted triangles) or CHIKV-3ʹ-Cre (open red triangles, also shown in Fig 4C) from 1 to 112 dpi. Each time point for each virus represents 6–20 mice. **(E)** Representative images of connective tissues in the ipsilateral ankle of a mouse infected with CHIKV-3ʹ-Cre. Blue shows DAPI staining, and red is tdTomato; scale bar represents 1000 μm. In higher magnification inset images scale bars represent 100 μm; (A) is adipose tissue, (T) is tendon, (B) is bone, (M) is muscle, and (S) is synovium. Data from **A**, **B**, and **D** were pooled from at least two independent experiments. Data in **A** and **B** were analyzed with an ordinary one-way ANOVA using Tukey’s post-test. Data in **D** were analyzed with two-way ANOVA using Sidak’s post-est. All error bars indicate SEM. (*, *P* < 0.05; **, *P* < 0.01; ***, *P* < 0.001; ****, *P* < 0.0001).
(TIF)

S6 Fig Gating strategy for skin and ankle flow cytometry. tdTomato mice were infected with 10^5^ PFU of CHIKV-3ʹ-Cre (3ʹ-Cre), ipsilateral skin **(A-C)** or ankle **(D-F)** tissue were harvested at 28 dpi, and single cell suspensions were generated and analyzed by flow cytometry as described in Methods. Gating strategy showing subpopulations of live cells in tdTomato mice in the ipsilateral skin **(A)** and ankle tissue **(D)**. The percentages of tdTomato^+^ fibroblasts were determined using CD45^-^ as well as CD29^+^ and CD44^int^. **(B, E)** Representative histogram of the tdTomato mean florescence intensity (MFI) for both the CD45^+^ and CD45^-^ cells in the ipsilateral skin **(B)** and ankle **(E)**. **(C, F)** Quantification of paired tdTomato MFI data for the CD45^+^ and CD45^-^ cells in the ipsilateral skin **(C)** and ankle **(E)**. Data represents three **(D-F)** or five **(A-C)** independent experiments. Data in **C** and **F** were analyzed using a paired Student’s *t* test. (*, *P* < 0.05).
(TIF)
