# Supplementary figures and images for: Dermal and muscle fibroblasts and skeletal myofibers survive chikungunya virus infection and harbor persistent RNA
Source: PLoS Pathog. 2019 Aug 29;15(8):e1007993. doi: 10.1371/journal.ppat.1007993 (PMC6715174; doi:10.1371/journal.ppat.1007993)

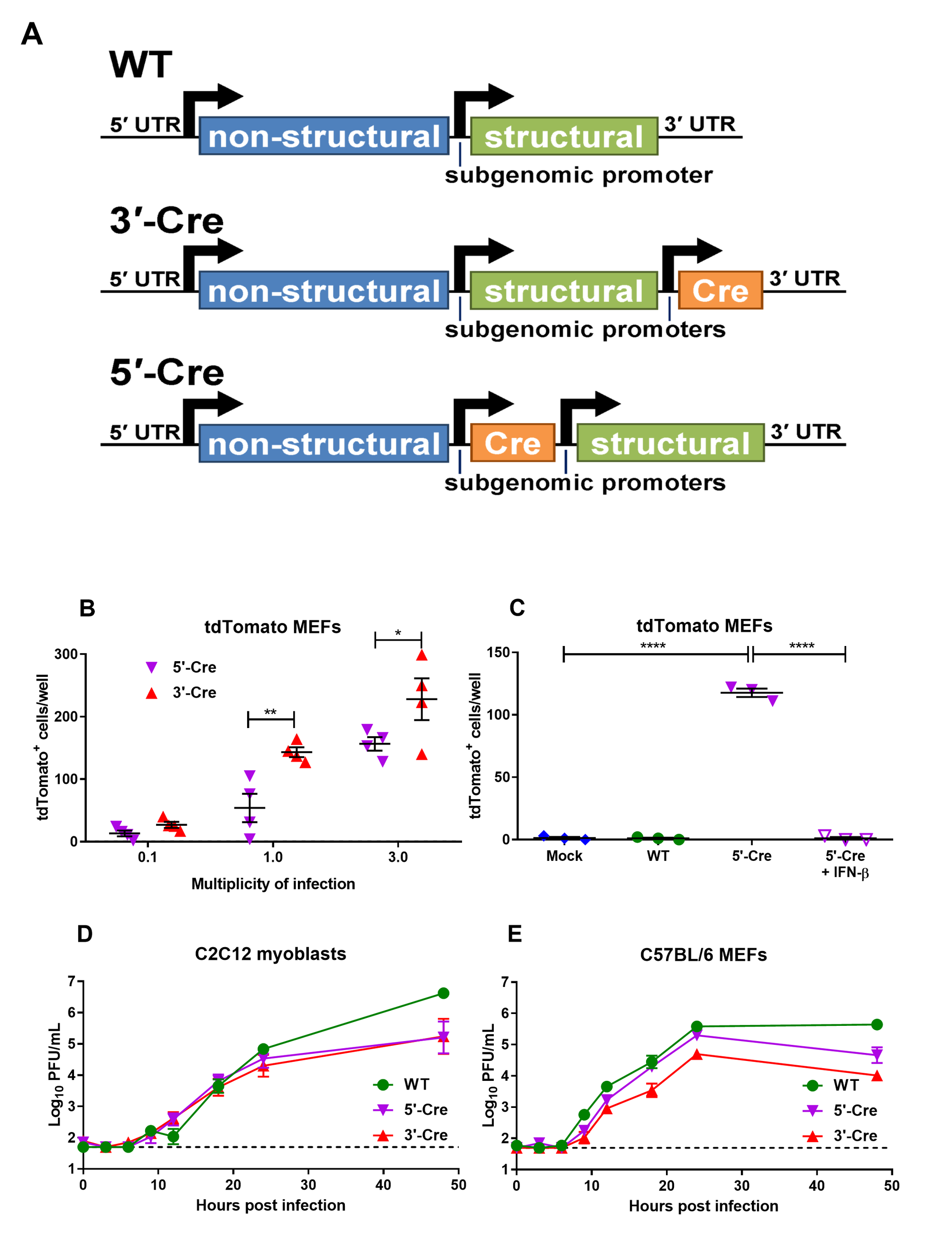

Supplement: S1 Fig — (A) Genome maps of the three CHIKV clones that were used in these studies: CHIKV-WT, CHIKV-3ʹ-Cre, and CHIKV-5ʹ-Cre. (B) tdTomato MEFs plated in 96-well plates (2.5 x 10^4^ cells/well) were inoculated with CHIKV-5ʹ-Cre (purple inverted triangles) or CHIKV-3ʹ-Cre (red triangles) at an MOI of 0.1, 1.0, or 3.0 and analyzed for fluorescence. (C) tdTomato MEFs plated in 96-well plates (2.5 x 10^4^ cells/well) and were mock-infected (mock) or infected at an MOI of 3.0 with CHIKV-WT (WT), CHIKV-5ʹ-Cre (5ʹ Cre), or CHIKV-5ʹ-Cre pretreated with ~100 U IFN-β (5ʹ Cre + IFN-β). (D) Representative growth curves of C2C12 myoblasts or (E) C57BL/6 MEFs infected with CHIKV-WT (green circles), CHIKV-5ʹ-Cre (purple inverted triangles), or CHIKV-3ʹ-Cre (red triangles) at an MOI of 0.05. The number of tdTomato+ cells per well in B and C were quantified at 2 dpi manually using a fluorescent microscope and are representative of two independent experiments. Data in B were analyzed with a two-way ANOVA using Sidak's post-test. Data in C were analyzed with an ordinary one-way ANOVA using Sidak’s post-test. All error bars indicate SEM. (*, P < 0.05; **, P < 0.01; ***, P < 0.001; ****, P < 0.0001). (TIF) [file ppat.1007993.s001.tif]

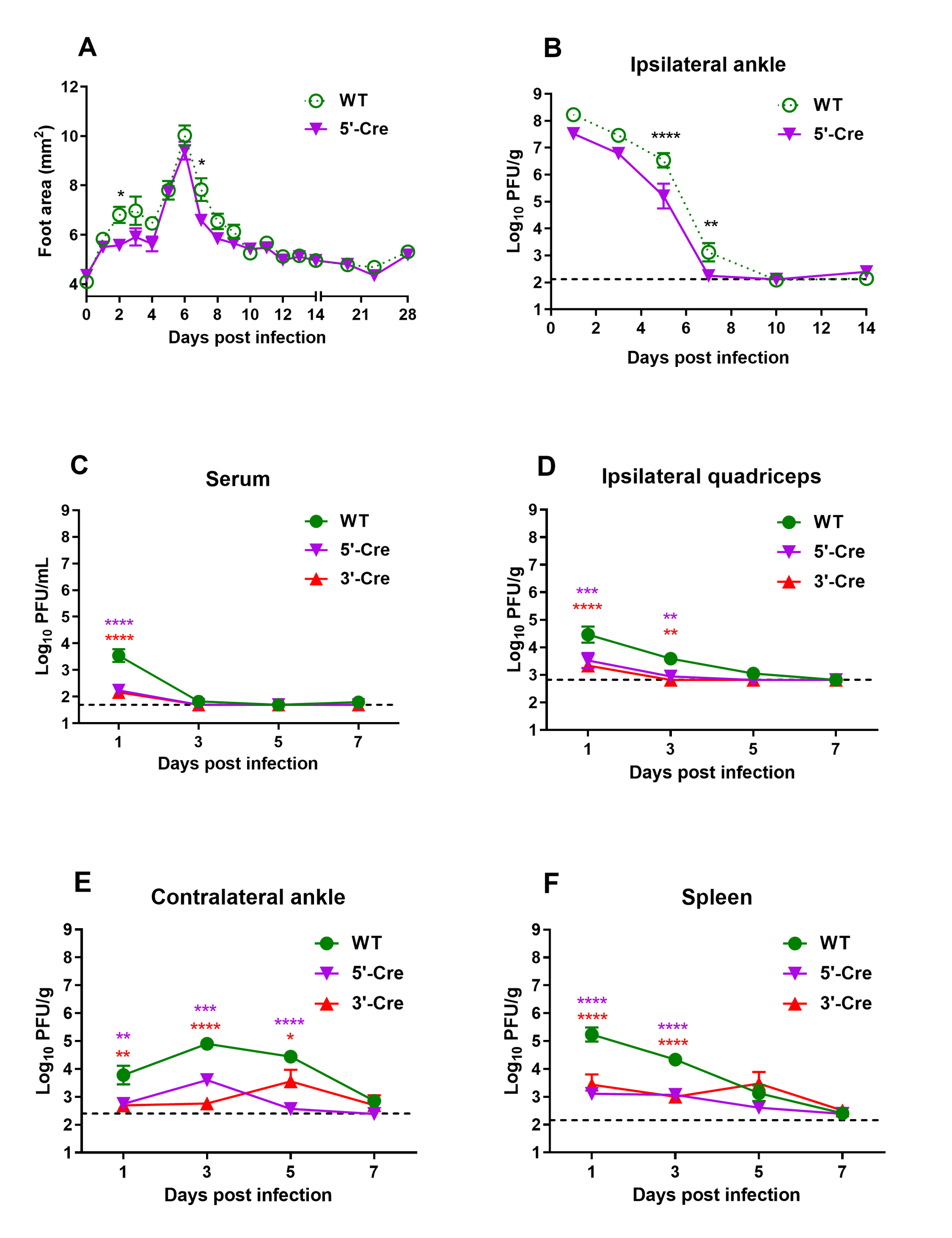

Supplement: S2 Fig — (A) Swelling of ipsilateral feet of mice inoculated with 106 PFU of CHIKV-WT (open green circles; also shown in Fig 2A) or 106 PFU of CHIKV-5ʹ-Cre (purple). Data were pooled from two independent experiments with n = 10 for each virus. (B-F) Levels of infectious virus in mice infected with 106 PFU of CHIKV-WT (solid green circles; or open green circles in S2B, also shown in Fig 2B), 106 PFU of CHIKV-5ʹ-Cre (purple inverted triangles), or 106 PFU of CHIKV-3ʹ-Cre (red triangles) in (B) ipsilateral ankle, (C) serum, (D) ipsilateral quadriceps muscle, (E) the contralateral ankle, or (F) spleen. For B-F, each time point for each virus and organ represents 5–7 mice and were pooled from at least 2 independent experiments. Infectious virus levels during acute infection was measured by plaque assay, normalized to gram of tissue, and then log-transformed. The dashed line for B-F represents limit of detection for the plaque assay. Data in B-F were log-transformed prior to analysis. Data in A were analyzed with a two-way repeated measures (RM) ANOVA with Bonferroni’s post-test, and data in B-F were analyzed with an ordinary two-way ANOVA. Sidak's post-test was used for A, and B; Dunnett's post-test comparing WT as the control column was used for C-F. All error bars indicate SEM. (*, P < 0.05; **, P < 0.01; ***, P < 0.001; ****, P < 0.0001). (TIF) [file ppat.1007993.s002.tif]

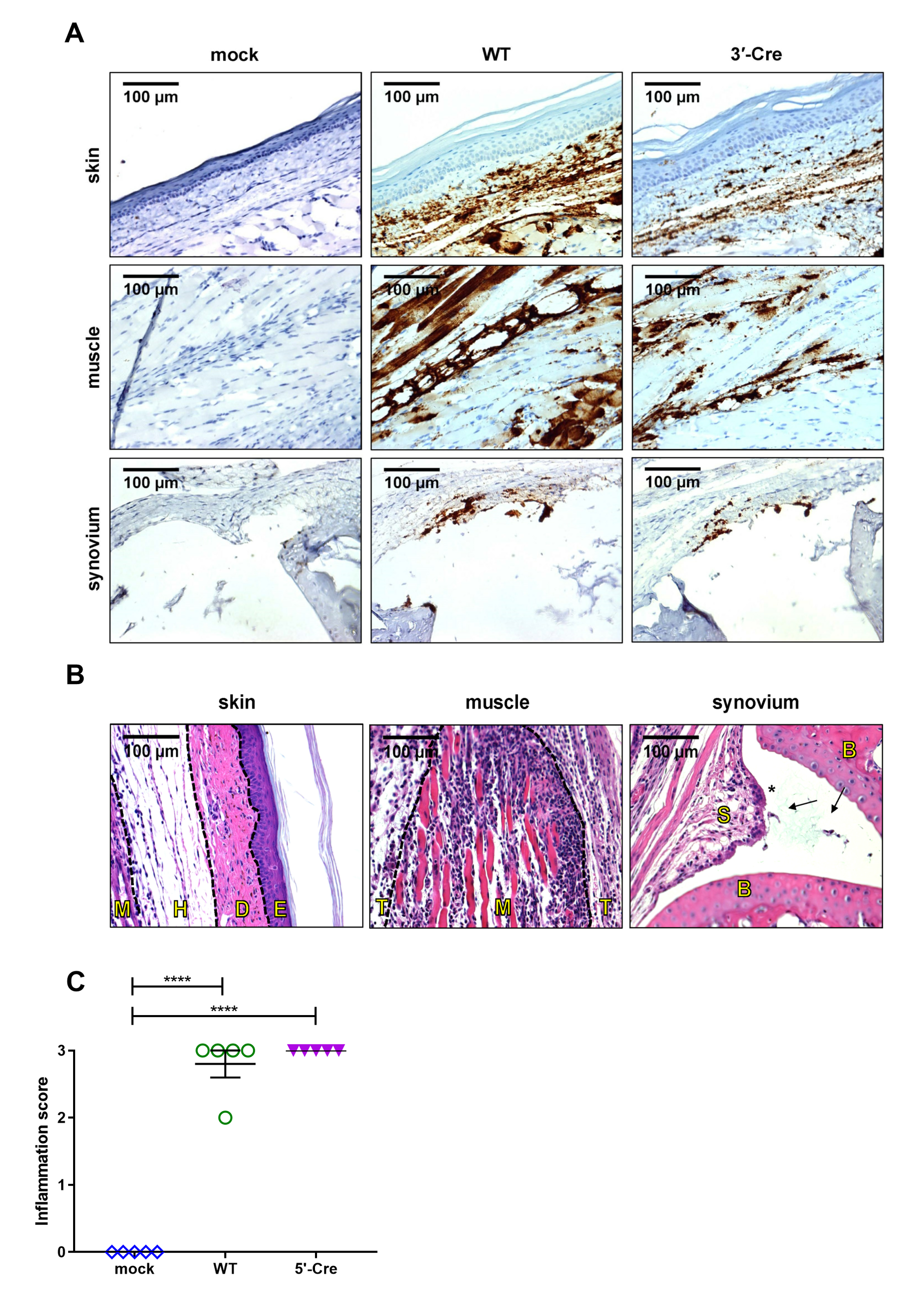

Supplement: S3 Fig — (A) Mice were mock-infected or infected with 106 PFU CHIKV-WT (WT) or CHIKV-3ʹ-Cre (3ʹ-Cre), and ipsilateral ankles were probed for CHIKV RNA using in situ hybridization at 2 dpi. Paraffin sections were stained with a probe for E1 CHIKV-LR RNA as outlined in the Methods. Representative images are shown of the skin, muscle, and synovium. Scale bars represent 100 μm. Data represents two independent experiments with 6 mice per virus and 2 mock-infected mice. (B-C) Mice were mock-infected (mock, blue diamonds) or inoculated with 106 PFU CHIKV-WT (WT, green circles) or CHIKV-5ʹ-Cre (5ʹ-Cre, purple inverted triangles), and ipsilateral ankles were taken for H&E histology at 7 dpi. (B) Representative images are shown of the skin, muscle, and synovium from CHIKV-5ʹ-Cre infected samples; scale bar represents 100 μm. The skin and associated tissue is divided (from left to right) into muscle (M), hypodermis (H), dermis (D), and epidermis (E). The muscle section is divided into tendon (T) and muscle (M). The synovium section shows synovium (S) and bone (B), with asterisks indicating synovial inflammation and arrows indicating immune infiltrates into the synovial cavity. (C) Ankles from B were scored for overall histological damage, compared to mock-infected and CHIKV-WT-infected samples. Open symbols for mock and WT indicate that these data are also shown in the corresponding Fig 2C graph. Samples were pooled from two independent experiments. Data in C were statistically analyzed with a one-way ANOVA with Tukey's post-test. All error bars indicate SEM. (*, P < 0.05; **, P < 0.01; ***, P < 0.001; ****, P < 0.0001). (TIF) [file ppat.1007993.s003.tif]

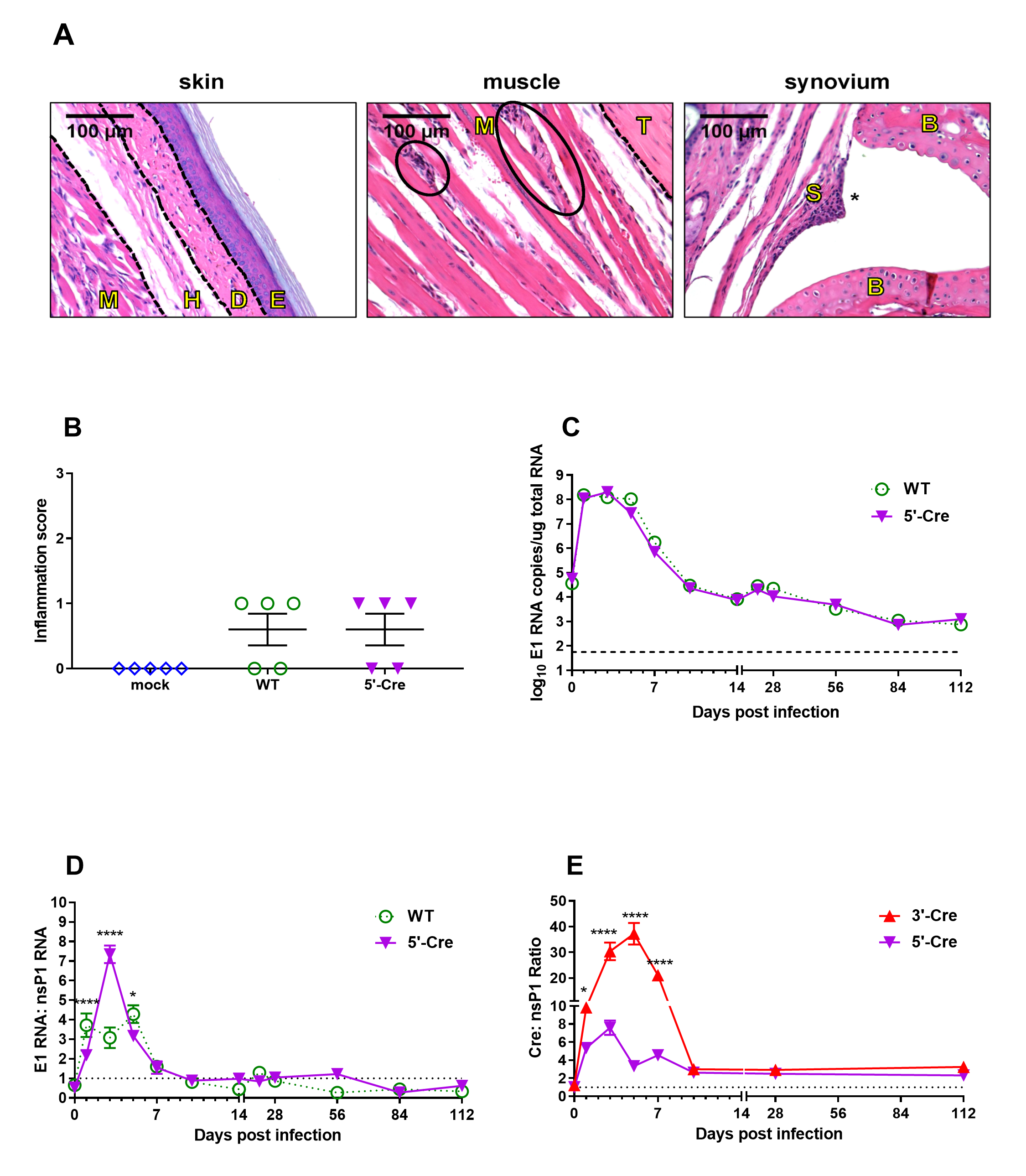

Supplement: S4 Fig — (A-B) Mice were mock-infected (mock, blue diamonds) or infected with 106 PFU CHIKV-WT (WT, green circles) or CHIKV-5ʹ-Cre (5ʹ-Cre, purple inverted triangles), and at 28 dpi ipsilateral ankles were analyzed histologically after hematoxylin and eosin staining of sections. (A) Representative images are shown of the skin, muscle, and synovium from CHIKV-5ʹ-Cre samples; scale bar represents 100 μm. The skin and associated tissue is divided (from left to right) into muscle (M), hypodermis (H), dermis (D), and epidermis (E). The muscle section is divided into muscle (M) and tendon (T), with black ovals around focal patches of cellular filtrates. The synovium section shows synovium (S) and bone (B), with asterisks indicating synovial inflammation and proliferation. (B) Ankles from A were scored for overall histological damage, compared to mock-infected and CHIKV-WT-infected samples. Open symbols for mock and WT indicate that these data are also shown in Fig 3B graph. (C) RNA was isolated from the ipsilateral ankles of mice inoculated with 106 PFU of CHIKV-WT (open green circles, also shown in Fig 3D) or 106 PFU of CHIKV-5ʹ-Cre (purple inverted triangles) at time points ranging from 0 to 112 dpi and analyzed by RT-qPCR for viral E1, nsp1, or Cre RNA copy number. Samples were normalized to total μg of RNA isolated for each sample and then log-transformed. (C) Viral E1 RNA levels were measured. (D) The ratio of viral E1 RNA to viral nsP1 RNA in the same samples as C (open green circles, also shown in Fig 3E). (E) The ratio of viral Cre RNA to viral nsP1 RNA in mice inoculated with 106 PFU of CHIKV-5ʹ-Cre (purple inverted triangles) or CHIKV-3ʹ-Cre (red triangles). Data in B were analyzed with a one-way ANOVA with Tukey's post-test. For C-E, each time point for each virus represents 4–20 mice and was pooled from at least 2 independent experiments. The dashed line in C represents the limit of detection for the RT-qPCR assay; for D and E, the dotted line represents a ratio of 1. [file ppat.1007993.s004.tif]

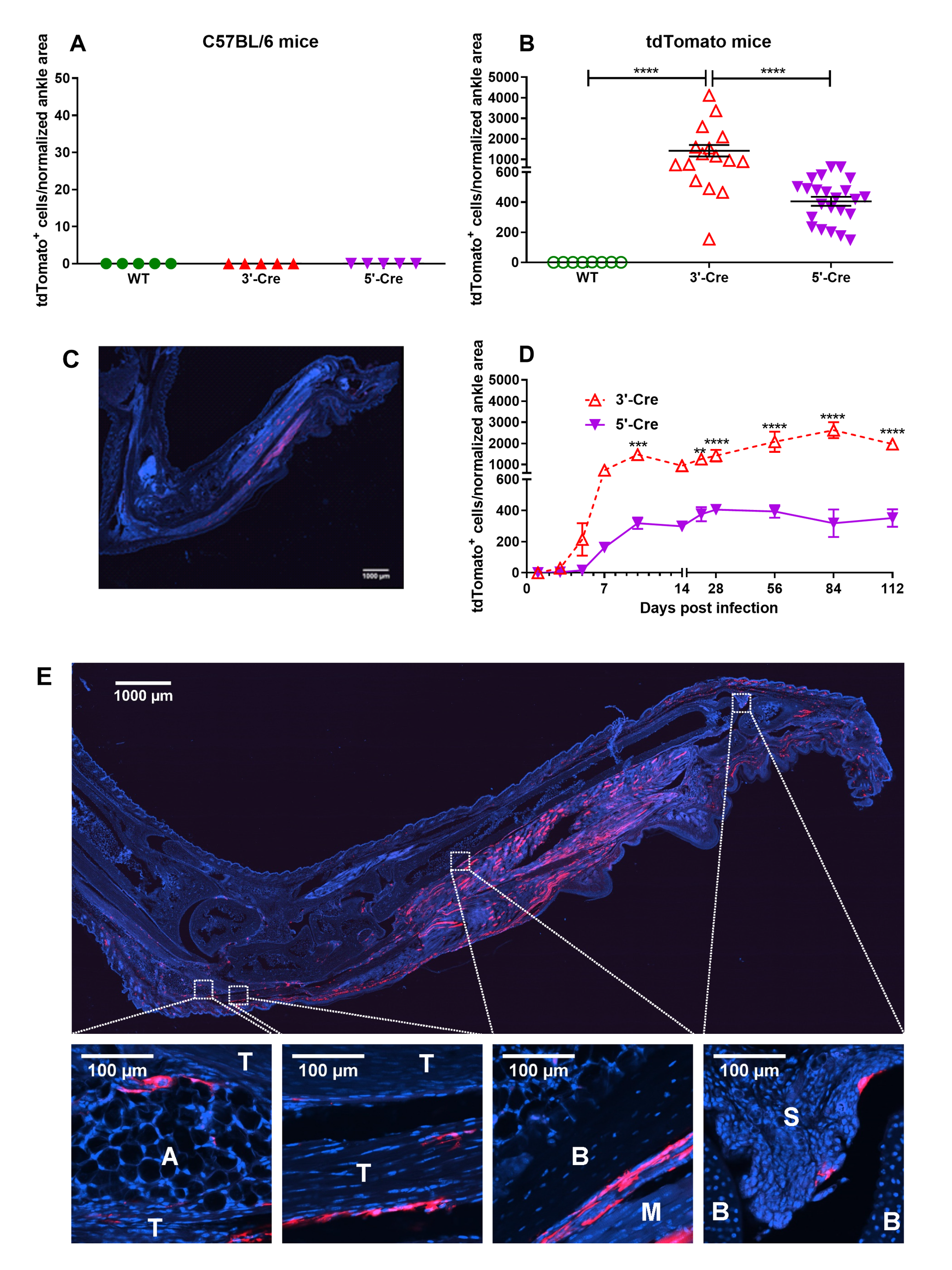

Supplement: S5 Fig — (A) C57BL/6 or (B) tdTomato mice were inoculated with 106 PFU CHIKV-WT (WT, green circles), CHIKV-5ʹ-Cre (5ʹ-Cre, purple inverted triangles), or CHIKV-3ʹ-Cre (3ʹ-Cre, red triangles). Ipsilateral ankles were processed at 28 dpi, and the total number of tdTomato+ cells in frozen tissue sections was quantified. Open green circles for WT and open red triangles for 3ʹ-Cre indicate that these data are also shown in the corresponding Fig 4B graph. (C) Representative image of a whole foot/ankle from a tdTomato mouse infected with 5ʹ-Cre at 28 dpi. Blue shows DAPI staining, and red is tdTomato; scale bars represent 1000 μm. (D) Time course of tdTomato mice infected with CHIKV-5ʹ-Cre (purpled inverted triangles) or CHIKV-3ʹ-Cre (open red triangles, also shown in Fig 4C) from 1 to 112 dpi. Each time point for each virus represents 6–20 mice. (E) Representative images of connective tissues in the ipsilateral ankle of a mouse infected with CHIKV-3ʹ-Cre. Blue shows DAPI staining, and red is tdTomato; scale bar represents 1000 μm. In higher magnification inset images scale bars represent 100 μm; (A) is adipose tissue, (T) is tendon, (B) is bone, (M) is muscle, and (S) is synovium. Data from A, B, and D were pooled from at least two independent experiments. Data in A and B were analyzed with an ordinary one-way ANOVA using Tukey’s post-test. Data in D were analyzed with two-way ANOVA using Sidak’s post-est. All error bars indicate SEM. (*, P < 0.05; **, P < 0.01; ***, P < 0.001; ****, P < 0.0001). (TIF) [file ppat.1007993.s005.tif]

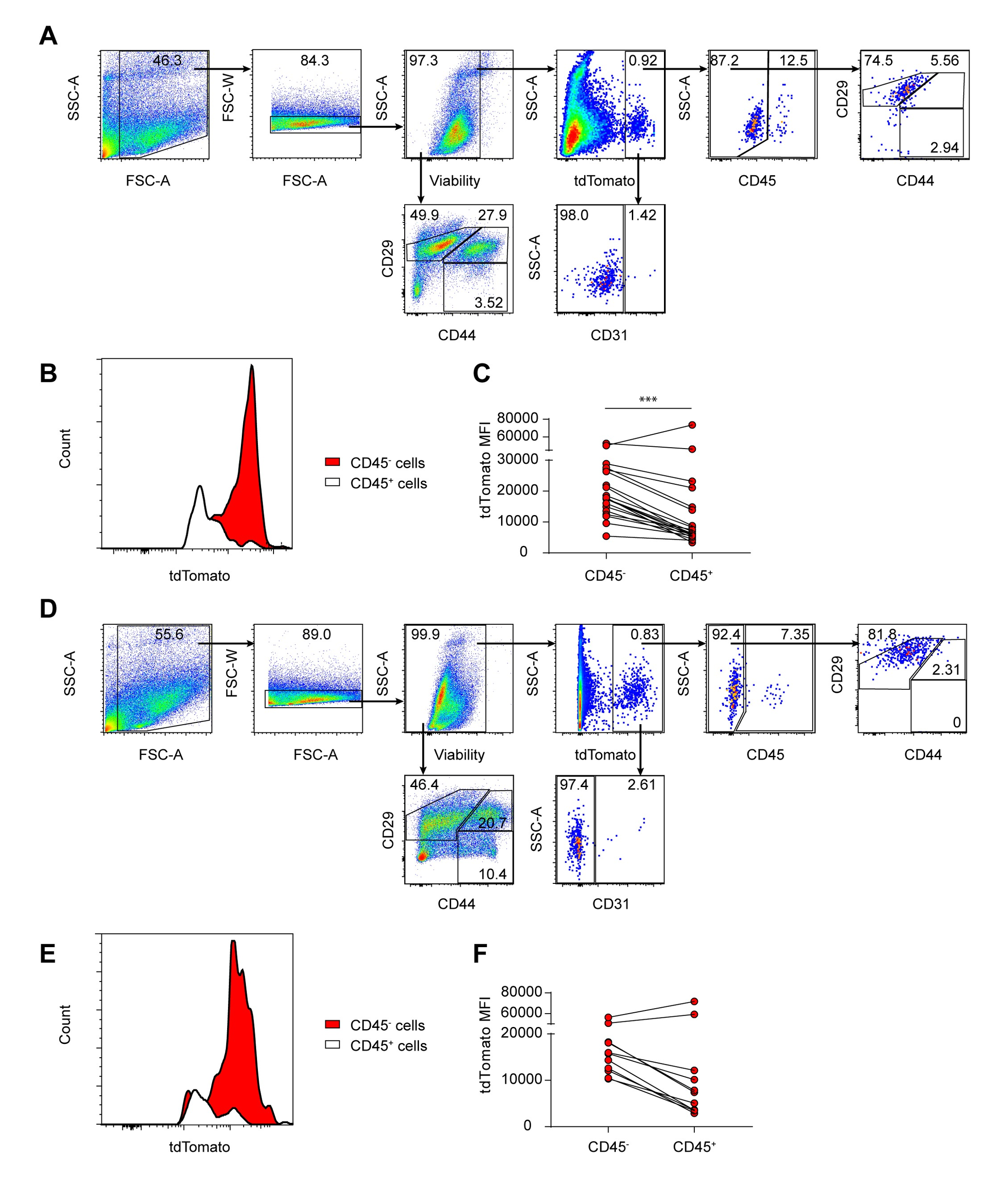

Supplement: S6 Fig — tdTomato mice were infected with 105 PFU of CHIKV-3ʹ-Cre (3ʹ-Cre), ipsilateral skin (A-C) or ankle (D-F) tissue were harvested at 28 dpi, and single cell suspensions were generated and analyzed by flow cytometry as described in Methods. Gating strategy showing subpopulations of live cells in tdTomato mice in the ipsilateral skin (A) and ankle tissue (D). The percentages of tdTomato+ fibroblasts were determined using CD45- as well as CD29+ and CD44int. (B, E) Representative histogram of the tdTomato mean florescence intensity (MFI) for both the CD45+ and CD45- cells in the ipsilateral skin (B) and ankle (E). (C, F) Quantification of paired tdTomato MFI data for the CD45+ and CD45- cells in the ipsilateral skin (C) and ankle (E). Data represents three (D-F) or five (A-C) independent experiments. Data in C and F were analyzed using a paired Student’s t test. (*, P < 0.05). (TIF) [file ppat.1007993.s006.tif]
